# OsNAC109 regulates senescence, growth and development by altering the expression of senescence- and phytohormone-associated genes in rice

**DOI:** 10.1007/s11103-021-01118-y

**Published:** 2021-02-04

**Authors:** Liangjian Li, Yan He, Zhihong Zhang, Yongfeng Shi, Xiaobo Zhang, Xia Xu, Jian-li Wu, Shaoqing Tang

**Affiliations:** State Key Laboratory of Rice Biology, China National Rice Research Institute, 359 Tiyuchang Road, Hangzhou, 310006 China

**Keywords:** Rice, Transcription factor, OsNAC109, Chlorophyll, Plant hormone, Senescence-associated gene

## Abstract

**Key message:**

We demonstrate that OsNAC109 regulates senescence, growth and development via binding to the cis-element CNTCSSNNSCAVG and altering the expression of multiple senescence- and hormone-associated genes in rice.

**Abstract:**

The NAC family is one of the largest transcripton factor families in plants and plays an essential role in plant development, leaf senescence and responses to biotic/abiotic stresses through modulating the expression of numerous genes. Here, we isolated and characterized a novel *yellow leaf 3* (*yl3*) mutant exhibiting arrested-growth, increased accumulation of reactive oxygen species (ROS), decreased level of soluble proteins, increased level of malondialdehyde (MDA), reduced activities of ROS scavenging enzymes, altered expression of photosynthesis and senescence/hormone-associated genes. The yellow leaf and arrested-growth trait was controlled by a single recessive gene located to chromosome 9. A single nucleotide substitution was detected in the mutant allele leading to premature termination of its coding protein. Genetic complementation could rescue the mutant phenotype while the *YL3* knockout lines displayed similar phenotype to WT. *YL3* was expressed in all tissues tested and predicted to encode a transcriptional factor OsNAC109 which localizes to the nucleus. It was confirmed that OsNAC109 could directly regulate the expression of *OsNAP*, *OsNYC3*, *OsEATB*, *OsAMTR1*, *OsZFP185*, *OsMPS* and *OsGA2ox3* by targeting to the highly conserved cis-element CNTCSSNNSCAVG except *OsSAMS1*. Our results demonstrated that *OsNAC109* is essential to rice leaf senescence, growth and development through regulating the expression of senescence- and phytohormone-associated genes in rice.

**Supplementary Information:**

The online version contains supplementary material available at 10.1007/s11103-021-01118-y.

## Introduction

Senescence is the final stage of plant development and is a tightly controlled genetic process at the organismal, celluar and molecular levels. In this process, the nutrients invested in aging tissues such as nitrogen, phosphorus and metals are reallocated to vigorously growing tissues and organs for reuse. Thus, leaf senescence can be viewed as a recycling program in the organism (Himelblau 2000). At the celluar level, leaf senescence is a form of programed cell death (PCD) when cells suffered massive changes in an orderly manner and one of its most distinctive characteristics is chloroplast degeneration with chlorophyll breakdown. The program leads to the first visible phenotypic change i.e. leaf yellowing. The leaf premature senescence means the shortening of crop growth stage due to the proceeding of chloroplast degeneration, an unfavorable state to agronomic production, although senescence is an active process to salvage nutrients from old tissues (Woo et al. 2013). Additionally, senescence as one type of PCD, is usually promoted by reactive oxygen species (ROS) accumulation and DNA degradation, whereas ROS scavenging enzymes, including superoxide dismutase (SOD), peroxidase (POD), and catalase (CAT) play improtant roles in regulating leaf senescence (He et al. 2018).

Although leaf senescence is an age-dependent process, it involves intricate and complex pathways that respond to various endogenous factors such as phytohormones and metabolism (Zhao et al. 2019), as well as exogenous factors including temperature, light, drought, nutrient deficiency, wounding, and pathogen infection (Yang et al. 2016; Wang et al. 2019). To explore the mechanisms behind leaf senescence, one of the best approaches is isolation and analysis of early senescence or delayed senescence mutants (Piao et al. 2019). To date, more than 800 senescence-associate genes (SAGs) have been identified, and genetic analysis reveals that leaf senescence is controlled by various negative and positive genetic elements (Lim et al. 2007).

Among SAGs isolated so far, the NAC (NAM, ATAF1/2, and CUC2) transcription factor (TF) family is important to modulate the process of leaf senescence through regulating gene expression. NAC TFs possess conserved NAC domains responsible for DNA binding, and highly variable C-terminal domains determining the transcription activity (Olsen et al. 2005b). The NAC domain consists of five subdomains (A–E), among which the highly conserved subdomains C and D are involved in DNA binding, whereas the divergent subdomains B and E are related to functional diversity of NAC TFs. The subdomain A may be responsible for dimerization (Ernst et al. 2004; Olsen et al. 2005a; Balazadeh et al. 2010; Kjaersgaard et al. 2011; Li et al. 2018). As for the NAC recognition sequence (NACRS), the Arabidopsis ANAC1 is able to bind to a 21 bp segment (CTGACGTAAGGGATGACGCAC) within the 35S-90 promoter region (Xie et al. 2000). ANAC019, ANAC055, and ANAC072 have been shown to recognize the sequence containing CATGT and CACG elements in vivo and in vitro (Tran et al. 2004). DAP-seq analysis, a TF-DNA binding assay in vitro, indicates potential NACRS of different NAC TFs in Arabidopsis, most of which contain highly conserved CNT and ANG elements (O’Malley et al. 2016). As closely-related NAC TFs show different preferences for the core binding site, the NACRS of a specific NAC TF needs further identification.

As one of largest the TF families, NAC family exists in most plant species including crops and trees, suggesting their fundamental role in plant growth and development. In Arabidopsis, AtNAC1 may activate the auxin-responsive genes, *DBP* and *AIR3*, to promote lateral root formation (Xie et al. 2000); AtNAC2 is also able to enhance plant lateral root development by integrating environmental and endogenous stimuli (He et al. 2005). Several NAC TFs (*ANAC002*/*ATAF1*, *ANA016*, *ANAC019*, *ANAC029*/*NAP*, *ANAC046*, *ANAC055*, *ANAC072*/*RD26*, and *ANAC092*/*ORE1*) positively regulate leaf senescence as their null mutants exhibit a stay-green phenotype during senescence (Kim et al. 2009, 2013; Takasaki et al. 2015; Oda-Yamamizo et al. 2016), whereas *ANAC042*/*JUB1* and *ANAC083*/*VNI2* negatively regulate leaf senescence (Yang et al. 2011; Wu et al. 2012). To date, more than 150 NAC TFs have been characterized in rice and they are involved in regulation of different biological processes. For the regulation of leaf senescence, *ONAC106* acts as an inhibitor by directly modulating the expression of SAGs such as *OsSGR* and *OsNYC1* (Sakuraba et al. 2015). *OsNAP* and *OsNAC2* mediate leaf senescence by simultaneously regulating the expression of SAGs and genes associated with ABA metabolism (Liang et al. 2014; Mao et al. 2017; Shen et al. 2017). The expressions of both *OsNAC5* and *OsNAC6* are increased during leaf senescence (Sperotto et al. 2009; Nakashima et al. 2007).

In this study, we isolated and characterized a premature senescece rice mutant *yellow leaf 3* (*yl3*) with retarded growth, delayed-heading and dwarfism phenotype. We found that the *yl3* phenotype is controlled by a pair of recessive mutation resulting from a single nucleotide substitution in the 2th exon of *YL3. YL3* encods a NAC transcriptonal factor OsNAC109 and expresses ubiquitously in rice. OsNAC109 regulates senescence, growth and development by altering the expression of hormone and senescence-associated genes via binding to a conserved cis-element CNTCSSNNSCAVG.

## Materials and methods

### Palnt materials and growth conditions

The rice *yl3* mutant was isolated from an ethyl methanesulfonate (EMS)-induced rice mutant library of the indica rice cultivar, Zhongjian100 (wild-type, WT). After mutiple generations of selfing, the premature senescence phenotype of *yl3* was stably inherited in different enviroments. *yl3* was crossed with a japonica rice cultivar Moroberekan to generate F_1_ plants and F_2_ populations for genetic analysis and gene mapping. All individuals were grown in the paddy field under natural conditions in Hangzhou, Zhejiang province and Lingshui, Hainan province, China. All transgenic plants were grown in the green house and net house under natural conditions at the China National Rice Research Institute (CNRRI) in Hangzhou. In addition, the seedlings of WT and *yl3* were hydroponically cultured on one half Hoagland medium in the phytotron for 2 weeks at 30 °C, 14-h light (400 μmol m^−2^ s^−1^)/24 °C, 10-h dark cycle (Liang et al. 2014) for determination of hormone contents and expression analysis of *YL3*. The means from three biological replicates were used for analysis by Student’s *t* test.

### Measurement of chlorophyll content and photosynthetic rate

The chlorophyll was extracted from the uppermost leaves of *yl3* and WT and measured as previously descriobed by Wellburn (1994) and Kim et al. (2006). The OD values at 470, 645, 663 and 652 nm were determined with a SpectraMax i3x Multi-Mode Microplate Reader (MOLECULAR DEVICES, Sunnyvate, CA, USA).

At 9:00–11:00 am on a sunny day under the paddy field conditions, the net photosynthetic rate (*Pn*) of flag leaves was determined by a portable device L-6400XT (LI-COR, Lincoln, NB, USA) with photosynthetic photon flux density (PPFD) of 1500 μmol m^−2^ s^−1^ and reference CO_2_ of 400 μmol mol^−1^ in the cuvette. All experiments were repeated three times and the means from three biological replicates were used for anlysis by Student’s *t* test or Duncan’s test.

### Transmission electron microscopy and toluidine blue cell staining

For transmission electron microscopy (TEM), the fully expanded flag leaves of *yl3* and WT plants grown in the paddy field at the heading stage were taken and fixed with 2.5% glutaraldehyde in 0.1 M phosphate buffer (pH 7.2) for 16 h at 4 ℃ after vacuuming. The subsequent analysis was carried out according to the method described previously (Huang et al. 2016). The section samples were observed under a Tenai G2F20 transmission electron microscope at the College of Agriculture and Biotechnology, Zhejiang University.

For toluidine blue cell staining, the uppermost internodes of *yl3* and WT at the heading stage were collected and fixed with formalin-acetic-alcohol (FAA) fixative. The vertical sectioned samples were handled and observed as previously described ( He et al. 2018).

### Detection of hydrogen peroxide and superoxide radical

To determine the accumulation of hydrogen peroxide (H_2_O_2_) and superoxide radical (O_2_^−^), the uppermost leaves from *yl3* and WT grown in the paddy field at the tillering stage were collected and stained with 3,3-diaminobenzidine (DAB) and nitrotetrazolium blue chloride (NBT), respectively, according to the methods described by Wang et al. (2014). The pictures were recorded with a HP ScanJet G4010 scanner (HP, Shanghai, China).

### TUNEL assays

The uppermost leaves were taken from *yl3* and WT plants grown in the paddy field at the tillering stage for the terminal deoxynucleotidyl transferase-mediated dUTP nick end labeling (TUNEL) assay with a Fluorescein in Situ Cell Death Detection Kit (Roche, Basel, Switzerland). The procedure of sectioning was used according to the method described previously (He et al. 2018).

### Determination of senescence-related paramaters

To determine senescence-associated parameters, the uppermost leaves from *yl3* and WT grown in the paddy field at the tillering stage were collected and frozen imeadiately in liquid nitrogen. Senescence-related paramaters including the contents of H_2_O_2_, soluble proteins (SP) and malondialdehyde (MDA), as well as activities of reactive oxygen species (ROS) scavenging enzymes (catalase, CAT; peroxidase, POD and superoxide dismatase, SOD) were determined following the manusfacture’s instruction (Nanjing Jiancheng Bioengeering Research Institute, China). The means of three biological replicates were used for analysis by Student’s *t* test.

### Map-based cloning of *YL3*

The F_1_ plants generated from the cross of the female parent *yl3* and male parent Moroberekan were grown in the paddy field to determine the dominant/recessive nature of *yl3* mutant phenotype. An F_2_ population derived from a selfed F_1_ plant (*yl3*/Moroberekan) was used for gene mapping. Bulked segregant analysis was first used to rapidly locate the mutation on a chromosome. Equal amount of leaf blades from each of 10 wild type plants and 10 mutant type plants were collected for DNA extraction to form a wild-type DNA pool and a mutant DNA pool, respectively. DNA of the parents and F_2_ mutant-type individuals were extracted following the mini-preparation method (Lu and Zheng 1992). Simple sequence repeat (SSR) markers were obtained from the website (http://www.gramene.org/) while insertion/deletion (InDel) markers were designed using the Primer 5.0 after comparison of the sequences between the japonica cultivar and the indica cultivar in the public database: Ensembl Plants (http://plants.ensembl.org/index.html). The primers were synthesized by Tsingke Biotech Co. Ltd (Hangzhou, China) and listed in Table S4. PCR reaction and detection were carried out as described previously (Chen et al. 2019).

### Complementation, knockout, GUS and subcellular localization assays

For the functional complementation, a 5631 bp genomic DNA containing the 2774 bp entire coding sequence of *YL3*, the 2173 bp upstream and the 684 bp downstream sequences were amplified from WT with the YL3-com primers (Supplementary Table S4), and subsequently the *Kpn*I and *Sma*I double-digested PCR product was inserted into the binary vector pCAMBIA1300 to generate a new construct p1300-YL3.

For the knockout assay, two 19 bp target sequences (5′AAGACGCTGGTCTACTACCG3′, 5′CTGTTCGTCCTGAACCCGTT3′) from the NAC domain for sgRNA targeting were cloned into the CRISPR/cas9 expression vector to generate two new CRISPR/cas9-mediated consructs pCRISPR-YL3-1 and pCRISPR-YL3-2, respectively, according to the method described previously (Ma et al. 2015).

For the *YL3-GUS* transient expression analysis, a 2025 bp upstream sequence of *YL3* was amplified with the YL3-GUS primer pairs (Supplementary Table S4). The product was fused to the GUS reporter gene and inserted into pCAMBIA1381Z vector to generate a new construct p1381-YL3. The constructs for complementation, knockout, and transient expression analysis were introduced into the embrogenic calli induced from *yl3*, Kitaake and Nipponbare respectively by *Agrobacterium tumefaciens*-mediated transformation (Hiei and Komari 2008).

For subcellular localization of *YL3*, the *YL3* full-length coding sequence (CDS) and the N-terminus of *YL3* were amplified using YL3-GFP F/R and N-YL3-GFP primers (Supplementary Table S4). The amplified full-length CDS was fused to the N-terminus of GFP and driven by the CaMV 35S promoter in the transient expression vector PAN580 to generate a new construct PAN-F. The N-terminus of *YL3* was fused to the N-terminal of GFP and driven by the CaMV 35S promoter in the transient expression vector PAN580 to generate a new construct PAN-N. The constructs were transformed into the WT protoplasts according to the method described by Chen et al. (2010). The fluorescence was observed 48 h after transformation by a Zeiss lsm710 confocal laser scanning microscope (Carl Zeiss, Inc., Jena, Germany).

### Yeast transcriptional activation assay

The full-length CDS of *YL3*, N-terminus, NAC domain, C-terminus, and the GAL4 DNA-activation domain of pGADT7 (postive control) were fused to the GAL4 DNA-binding domain in pGBKT7 to generate pGBKT7-F, pGBKT7-N, pGBKT7-domain, pGBKT7-C, and pGBKT7-AD constructs which then were transformed into the yeast strain Y2HGold, respectively, according to the manufacturer’s instruction (Clontech, http://www.clontech.com). The pGBKT7 empty vector was used as a negative control. The transcriptional activation was evaluated according to the growth of transformants on synthetic dropout (SD) medium SD/-Trp and SD/-Trp/-His/-Ade supplemented with 5-Bromo-4-chloro-3-indoxyl-α-D-galactopyranoside, respectively. The primers used were listed in Supplementary Table S4.

### Yeast one-hybrid assay

The full-length CDS of *YL3* and the NAC domain were amplified and fused to the C-terminus of B42 transcriptional activitor from pB42AD to construct two effectors pB42AD-YL3 and pB42AD-NAC, respectively. For the reporter constructs, the promoters with approximate 2 kb size from different genes (including *OsSAMS1*, *OsNAP* and *OsNYC3*) were amplified and inserted in the placZi vector to drive the lacZ reporter. The effector and reporter constructs were transformed into the yeast strain EGY48 following the manufacturer’s instruction (Clontech, http://www.clontech.com). The transformants were firstly grown and selected on the SD/-Trp/-Ura medium, and the positive colonies were then transferred onto the SD/-Trp-Ura medium containg 5-bromo-4-chloro-3-indolyl-β-D-galactopyranoside for coloration (Kayani et al. 2019). The primers used are listed in Supplementary Table S4.

### Dual-luciferase assay

The promoter fragments of selected genes (including *OsSAMS1*, *OsNAP* and *OsNYC3*) were amplified and cloned into pGreenII 0800–LUC acting as reporters, respectively. The full-length CDS of *YL3* was ampilfied to replace the GFP coding sequence in the transient expression vector PAN580 to generate a new effector construct *35S::YL3*. The effector and reporter constructs were co-transformed in rice protoplasts generated from WT according to Chen et al. (2010). After 48 h transformation, firefly and *Renilla* luciferase activities were determined with a Dual-Luciferase reporter assay kit (Promega, WI, USA). The means from three biological replicates were used for analysis by Student’s *t* test. The primers used are listed in Supplementary Table S4.

### Electrophoretic mobility shift assays (EMSA)

The full-length CDS of *YL3* was amplified and cloned into the pGEX-4 T-1 vector to generate a new construct pGEX-YL3 which was tranformed into the *E.coli* strain BL21 by heatshock at 42 ℃ for 1 min. The protein expression was induced at 18 ℃ overnight with 0.1 mM IPTG. GST-YL3 fusion proteins were purified using the GST SefinoseTM Resin Kit according to the manufacturer’s instructions (Sangon Biotech, Shanghai, China). To perform electrophoretic mobility shift assay, the candidate binding sequences based on Arabidopsis AtNAC57 were labeled with biotin and incubated with the fusion protein GST-YL3 using a chemiluminescent EMSA Kit (Beyotime Biotechnology, Haimen, China).

### RNA extraction and gene expression analysis

For expression pattern analysis of *YL3*, the total RNA was extracted from the shoots and roots of WT at the seedling stage, as well as extracted from the spikelets, leaf blades, leaf sheaths, internodes, nodes, and roots of WT at the heading stage. For expression analysis of 4 senesence-associated genes and 12 photosynthesis-related genes, the total RNA was extracted from leaves of *yl3* and WT at the tillering stage. For expression analysis of 3 *YL3-*targeted genes and 12 phytohormone biosynthesis-related genes, the total RNA was extracted from the shoots of *yl3* and WT at the seedling stage. The hydroponically cultured seedlings and the leaves from the plants grown in the field at the tillering and heading stages were sampled and frozen immediately at − 80 °C for RNA isolation. Total RNA extraction was carried out using a NucleoZOL Reagent Kit (MACHEREY–NAGEL, Düren, Germany) following the manufacturer’s instructions. All the primers used for expression analysis are listed in Supplementary Table S4. The first strand of cDNA was synthezied with PrimeScript™RT Master Mix (Takara, Kusatsu, Japan) following the manufacturer’s protocol and quantitative real-time PCR (qRT-PCR) was carried out using the TB Green™ Premix Ex Taq™ II (Takara, Kusatsu, Japan) on a Thermal Cycle Dice Real Time System (Takara, Kusatsu, Japan). Rice *Ubiquitin* (*LOC_Os03g13170*) was used as an internal control. The means from three replicates were used for analysis and the relative transcript levels were calculated by the 2^−ΔΔCt^ method (Schmittgen and Livak 2008).

### Extraction and determination of hormone levels

The levels of indole-3-acetic acid (IAA), Zeatin, abscisic acid (ABA), Gibberellic gibberellin A3 (GA3) and 1-aminocyclopropane-1-carboxylic acid (ACC) from 2-week-old seedlings and heading plants of *yl3* and WT were determined by Zoonbio Biotechnology Co., Ltd, Nanjing, China. The hydroponically cultured seedlings and the leaves from plants grown in the field at the heading stage were sampled for hormone analysis. Among them, the levels of IAA, Zeatin, ABA and GA3 were determined following the method described by (Zhang et al. 2017). For ACC, approximately 0.5 g 2-week-old seedling samples were ground in liquid nitrogen and immediately transferred to 50 mL tube containing 5 mL deionized water, then ultrasonically extracted for 30 min. After centrifuging at 16,020×*g* for 5 min under 4 °C, the supernatant was collected and adjusted pH 4.0. Then, 20 mL dichloromethane was added and the sample was centrifuged at 16,020×*g* for 5 min under 4 °C. The supernatant was loaded onto a MCX column that was activated by 3 mL methanol and 3 ml deionized water, and then washed by 2 mL methanol and 1 mL deionized water. The MCX column was eluted using 5 mL 1 mM ammonium hydroxide and the eluates were filtered through a 0.22 μm filter membrane. The resulting sample solution was injected into the high-performance liquid chromatography-tandem mass spectrometry (HPLC–MS/MS) for analysis according to Zhang et al. (2017).

### Transicriptome analysis

To perform transcriptome analysis, *yl3* was backcrossed to WT and the F_1_ plants were backcrossed again to generate BC_2_F_2_. Three mutant type individuals from BC_2_F_2_ and three WT individuals at the tillering stage were selected and the middle parts of the uppermost leaves were sampled for isolation of total RNA. A total of 6 RNA samples were extracted and used for RNA-seq analyses following the method described by Zhao et al. (2019).

## Results

### Performance of *yl3*

**Fig. 1:**
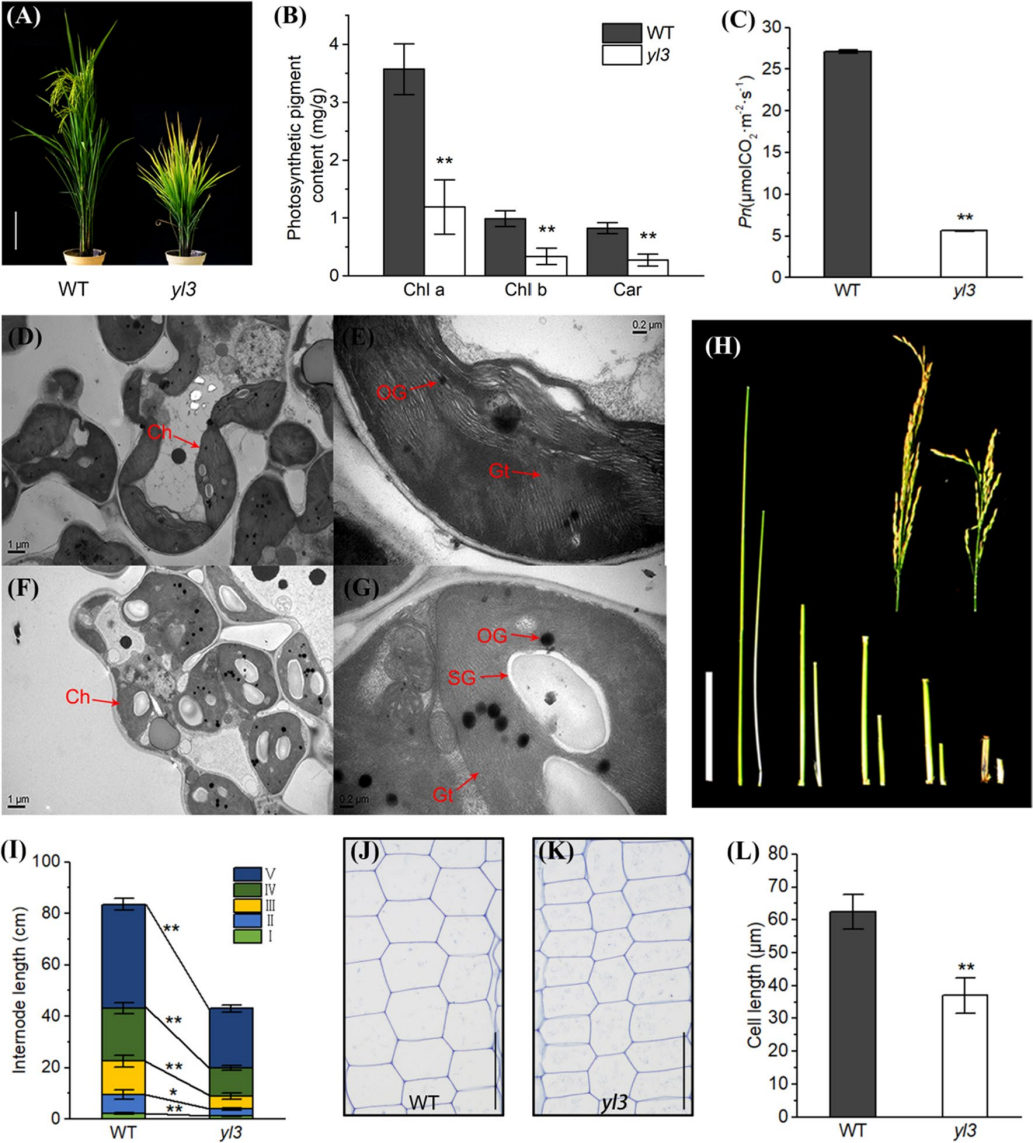
Characterization of *yl3* and the wild type. **a** Phenotype of *yl3* and WT at 80 days after sowing. Bar = 20 cm; **b** Photosynthetic pigment contents in WT and *yl3* at the tillering stage (means ± SD, *n* = 3); **c**. Net photosynthetic rate (*Pn*) of WT and *yl3* (means ± SD, *n* = 3); **d**–**g** Chloroplast ultrastructure of flag leaves in WT (**d**, **e**) and *yl3* (**f**, **g**). Ch, chloroplast; OG, osmiophilic granule; SG, starch granule; Gt, grana thylakoid; **h** The comparison of internode between WT and *yl3*; **i** Internode length of WT and *yl3* at the mature stage (means ± SD, *n* = 3); **j**, **k**. Longitudinal section of the uppermost internode of WT (**j**) and *yl3* (**k**), Bar = 100 μm; **l** Internode cell length of WT and *yl3* (means ± SD, *n* = 10). *P < 0.05, **P < 0.01 by Student’s *t* test

Under the field conditions, the leaf tips of *yl3* started to turn yellowish at the tillering stage and more than a half of the whole blade turned yellowish at the heading stage. At about 80 days after sowing (DAS), *yl3* showed arrested-growth with dwarfism and delayed heading date compared with WT (Fig. 1a). In addition, the lengths of shoots and roots of the 2-week-old seedlings of *yl3* under hydroponically cultured conditions were significantly lower than those of WT (Supplementary Fig. S1). The contents of chlorophyll a (Chl a), chlorophyll b (Chl b) and carotenoid (Car) in *yl3* at 60 DAS were decreased significantly compared with WT (Fig. 1b). Transmission electron microscopy (TEM) analysis indicated that the number and size of chloroplasts were reduced dramatically in the mesophyll cells of *yl3* compared to WT (Fig. 1d, f). In addition, more osmiophilic globuli, starch grains and impaired stromal lamellae were observed in chloroplasts of *yl3* suggesting the abnormal development of chloroplasts in *yl3* (Fig. 1e, g). Furthermore, the net photosynthetic rate of *yl3* at the heading stage was dramatically decreased compared with WT (Fig. 1c). The major agronomic traits of *yl3*, including plant height, panicle length, seed setting rate (No. filled grains/total no. grains per plant) and 1000-grain weight were significantly lower than those of WT at the mature stage (Supplementary Table S1). To determine the cause of dwarfism of *yl3*, we further measured the length of all internodes at the mature stage and found that the length of all the internodes in *yl3* was significantly shorter than that of WT (Fig. 1h, i). Further investigation of cell length in the internodes by toluidine blue staining suggested that the internode cell length of *yl3* was significantly shorter than that of WT (Fig. 1j-l), indicating that the dwarfism of *yl3* was due to the reduced internode cell length.

### *yl3* shows ROS-associated premature senescence

**Fig. 2:**
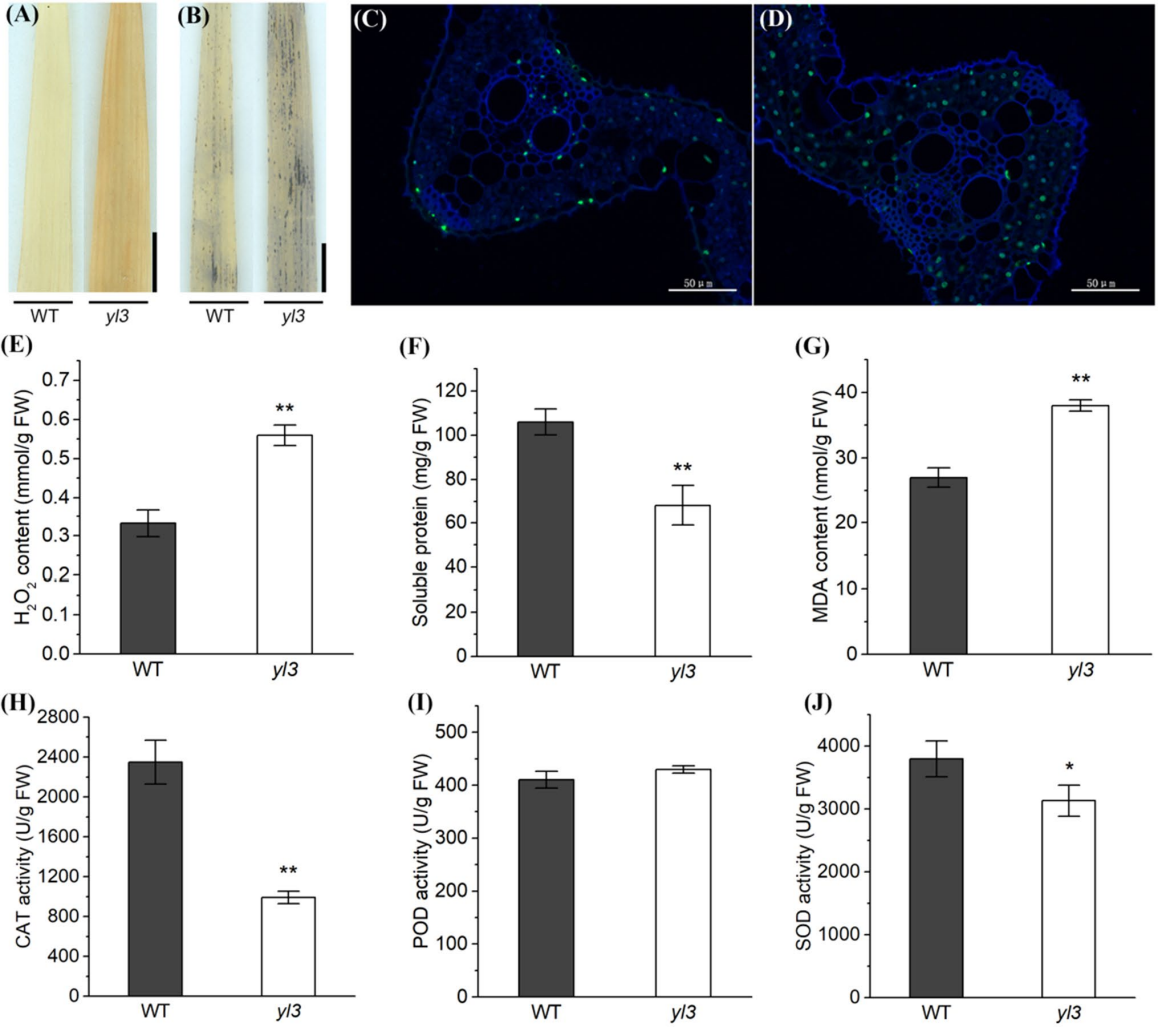
Histochemical analysis of physiological parameters related to senescence in WT and *yl3*. **a**, **b** DAB staining (**a**) and NBT staining (**b**) of the uppermost leaves in WT and *yl3*. Bar = 1 cm; **c**, **d** TUNEL assay of WT (**c**) and *yl3* (**d**); **e**–**g** The content of H_2_O_2_ (**e**) total soluble protein (**f)** and MDA (**g**) in WT and *yl3*; **h**–**j** The enzymatic activities of CAT (**h**), POD (**i**) and SOD (**j**) in WT and *yl3*. Values are means ± SD (*n* = 3). *P < 0.05, and **P < 0.01 by Student’s *t* test

To determine whether the yellowish leaf phenotype is associated with ROS accumulation in *yl3*, we carried out histochemical analysis. The results indicated that brown precipitates were observed in *yl3* compared with WT by DAB staining (Fig. 2a), and more blue formazan precipitates were observed in *yl3* than those of WT by NBT staining (Fig. 2b), indicating an elevated accumulation of H_2_O_2_ and O_2_^−^ in *yl3* (Fig. 2a, b). The endogenous H_2_O_2_ level in *yl3* was significantly higher than that of WT (Fig. 2e). The accumulation of ROS implied that the balance of ROS scavenging system was disrupted. The activities of CAT and SOD declined significantly in *yl3* whereas the activity of POD was similar compared with WT (Fig. 2h–j). Besides the changes of enzymatic activities, the expression of ROS-associated genes was apparently impacted in *yl3* at 3-week old seedlings (Supplementary Fig. S2). It was showed that most genes such as *NOX1*, *CATA*, *CATC* and *SODCC1* were up-regulated, while *SODB* was apparently down-regulated in *yl3* compared to WT. We further performed a TUNEL assay to determine DNA fragmentation and measured the total soluble protein content to determine the protein degradation. The results showed that more 4′, 6-diamino-phenylindole (DAPI) stained spots were found in *yl3* compared with WT (Fig. 2c, d), and the total soluble protein level in *yl3* was decreased markedly compared with WT (Fig. 2f). Furthermore, the MDA content was prominently increased in *yl3* compared to WT (Fig. 2g). Taken together, our results indicated that *yl3* was a premature senescence mutant in companion with an impaired ROS scavenging system.

### The mutation affects the expression of senescence- and photosynthesis-related genes

**Fig. 3:**
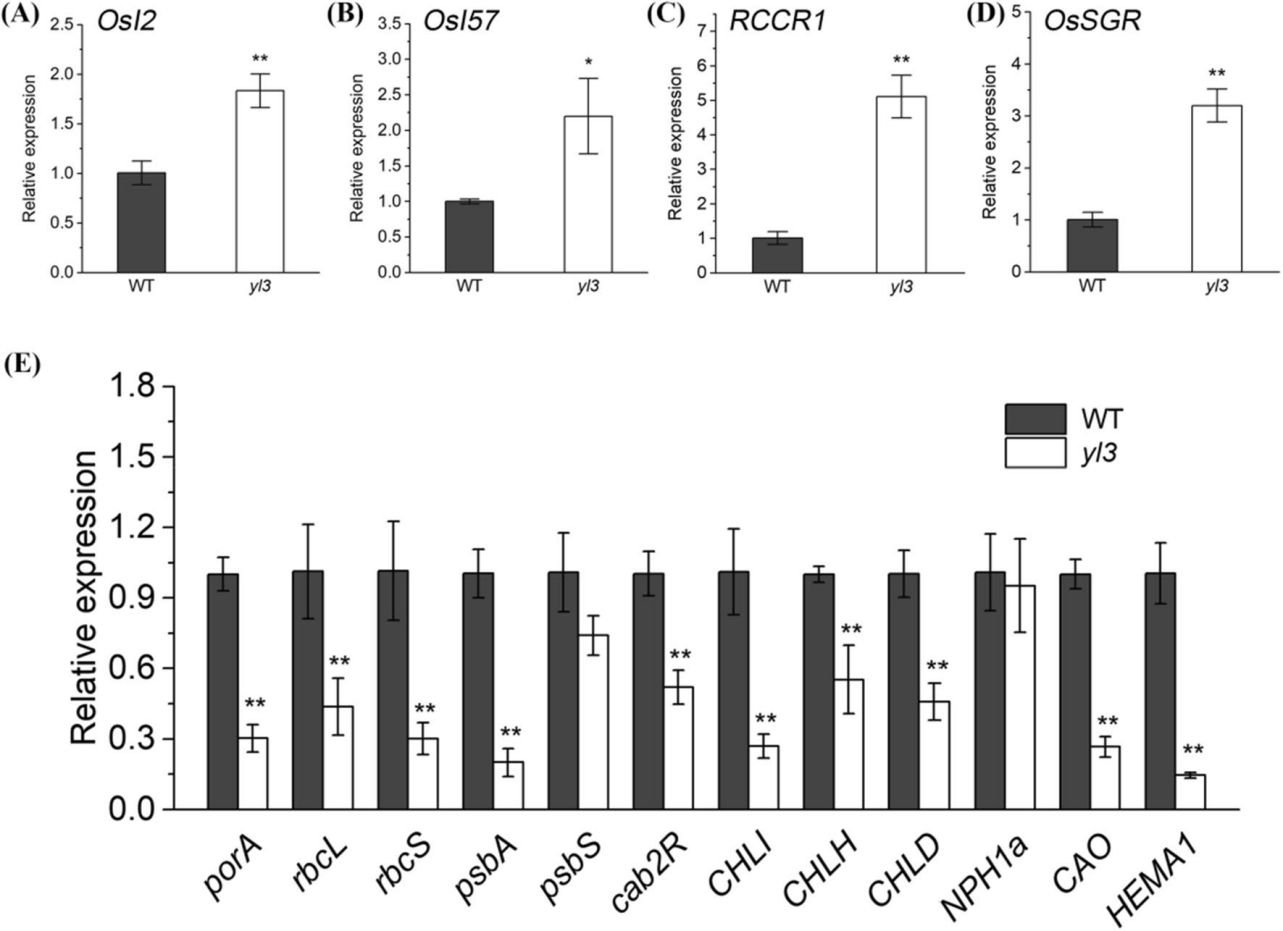
Expression of senescence- and photosynthesis-associated genes. **a–d** Expression levels of senescence-associated genes; **e** Expression levels of photosynthesis-related genes. Values are means ± SD (*n* = 3). *P < 0.05, and **P < 0.01 by Student’s *t* test

To reveal the impact of *yl3* mutation on leaf senescence and photosynthesis at the transcriptional level, we examined the expression of a set of selected genes, including senescence associated genes (SAGs) *OsI2*, *OsI57*, *RCCR*1 and *OsSGR;* photosynthetic regulatory genes *porA*, *rbcL*, *rbcS*, *psbA*, *psbS*, *cab2R*, *NPH1a* and *HEMA1*, and chlorophyll synthesis genes *CHLI*, *CHLD*, *CHLH* and *CAO*. The results showed that all the SAGs examined were up-regulated significantly in *yl3* (Fig. 3a–d), whereas the expression levels of chlorophyll synthesis genes were suppressed remarkably in *yl3* (Fig. 3e). The majority of photosynthesis-associated genes were apparently down-regulated except for *psbS* and *NPH1a* which were similar between *yl3* and WT (Fig. 3e). The results clearly demonstrated that *YL3* significantly down-regulated the expression of some SAGs, while mutation of *YL3* down-regulated the expression of photosynthesis associated genes.

### Map-based isolation of *YL3*

**Fig. 4:**
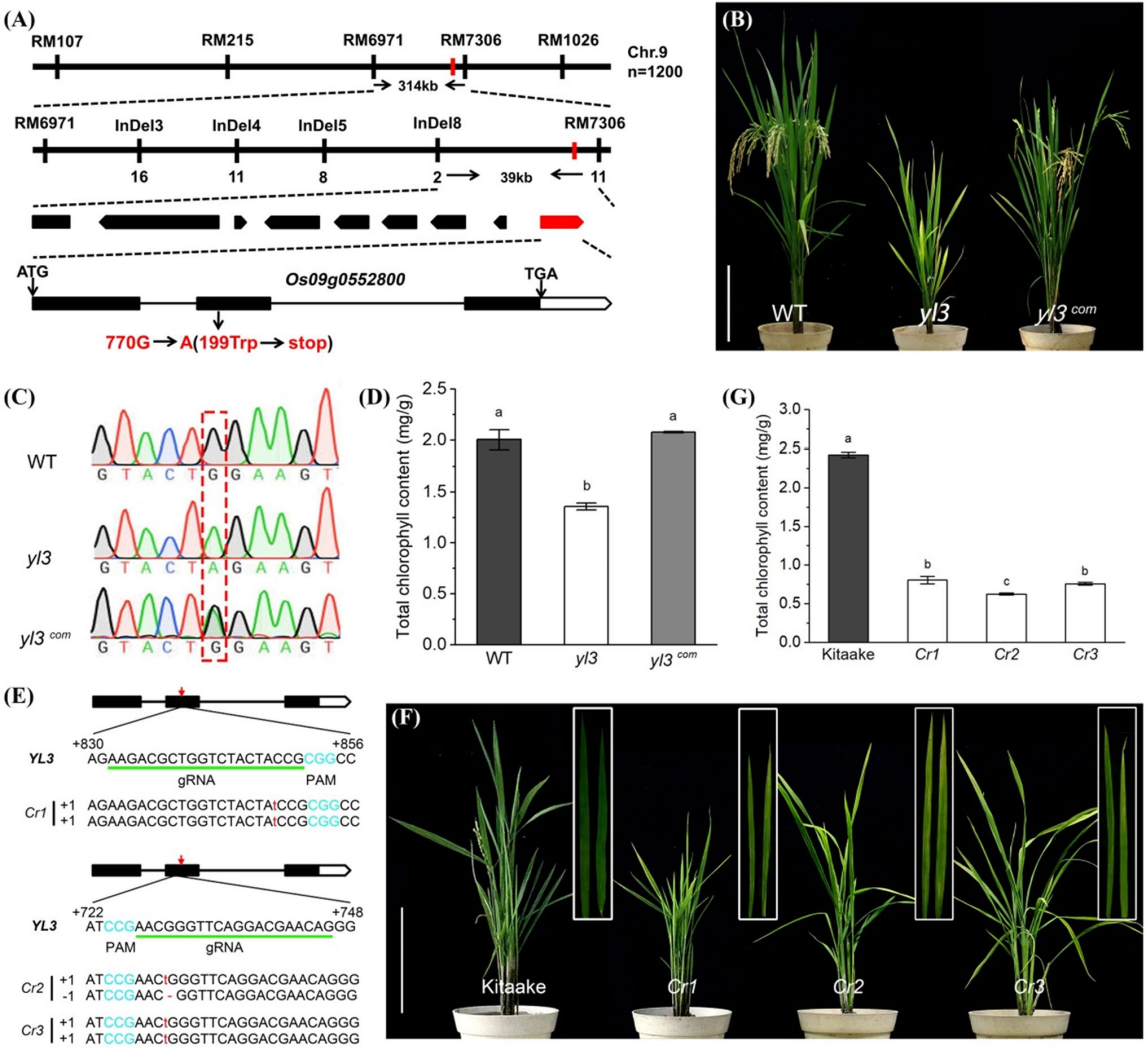
Map-base cloning and functional validation of *YL3*. **a** Fine mapping of *YL3*. The molecular markers and numbers of recombinants are indicated above and below the bars, respectively; **b** Functional complementation of *YL3*. Bar = 20 cm; **c** Comparison of the mutation sites among WT, *yl3* and complementary line *yl3*^*com*^; **d** Total chlorophyll content in WT, *yl3* and *yl3*^*com*^ (means ± SD, *n* = 3); **e** Mutations at the target sites in three representative knockout lines; **f** Phenotype of three representative knockout lines *Cr1*, *Cr2* and *Cr3*. Bar = 20 cm; **g** Total chlorophyll content in Kitaake and three representative knockout lines (means ± SD, *n* = 3). Different lowercase letters above the bars indicate a statistical difference at *P* ≤ 0.05 by one-way ANOVA and Duncan’s test

To isolate the gene responsible for the yellowish phenotype, we crossed *yl3* with the male parent Moroberekan and WT to generate F_1_ plants, respectively. All F_1_ individual plants from both crosses of *yl3*/Moroberekan and *yl3*/WT exhibited the normal green phenotype, indicating that the mutation was recessive in nature. However, in the field-grown F_2_ segregating population derived from *yl3*/Moroberekan, the normal green and yellowish plants did not show a single gene segregation ratio (3:1) or a double gene segregation ratio (15:1) (Supplementary Table S2). From the F_2_ population of *yl3/*Moroberekan, 1200 yellowish individual plants were selected for mapping by bulked segregant analysis. The *yl3* locus was initially mapped in the region flanked by markers RM6971 and RM7306 on the long arm of chromosome 9 and was further narrowed down to a 39 kb genomic region between InDel8 and RM7306. Nine open reading frames (ORFs) were identified based on the annotation from the RGAP databank (http://rice.plantbiology.msu.edu/index.shtml). Sequence comparison between *yl3* and WT revealed a single nucleotide substitution from G to A at the position 770 in the second exon of Os09g0552800, leading to a premature termination of the coding sequence (Fig. 4a). The results suggested that *Os09g0552800* is likely the candidate gene of *YL3* responsible for the yellowish phenotype.

To verify whether the mutation of *YL3* is responsible for the *yl3* phenotype, we transformed the construct p1300-YL3 into the *yl3-*derived calli through *A. tumafacien*-mediated transformation. A total of 12 transformants were obtained and all of them exhibited normal green phenotype similar to WT (Fig. 4b, c). In addition, the content of total chlorophyll, total soluble protein, MDA and H_2_O_2_ in the transformants recovered to the WT level (Fig. 4d; Supplementary Fig. S3a-c). Furthermore, the CRISPR/cas9-mediated constructs pCRISPR-YL3-1 and pCRISPR-YL3-2 targeting the conserved NAC domain of *YL3* were introduced to the embryogenic calli induced from the cultivar Kitaake. A total of 18 knockout lines were obtained and all of them exhibited a yellowish phenotype with reduced content of total chlorophylls and soluble proteins compared with the wild type Kitaake (Fig. 4e–g; Supplementary Fig. S3d). These results demonstrated that *Os09g0552800* was indeed the candidate gene of *YL3* responsible for the yellowish phenotype in *yl3*. *Os09g0552800* encodes a putative NAC (NAM, ATAF1/2, and CUC2) transcriptional factor 109 based on the RAP-DB databank (https://rapdb.dna.affrc.go.jp/).

### *YL3* is widely expressed

**Fig. 5:**
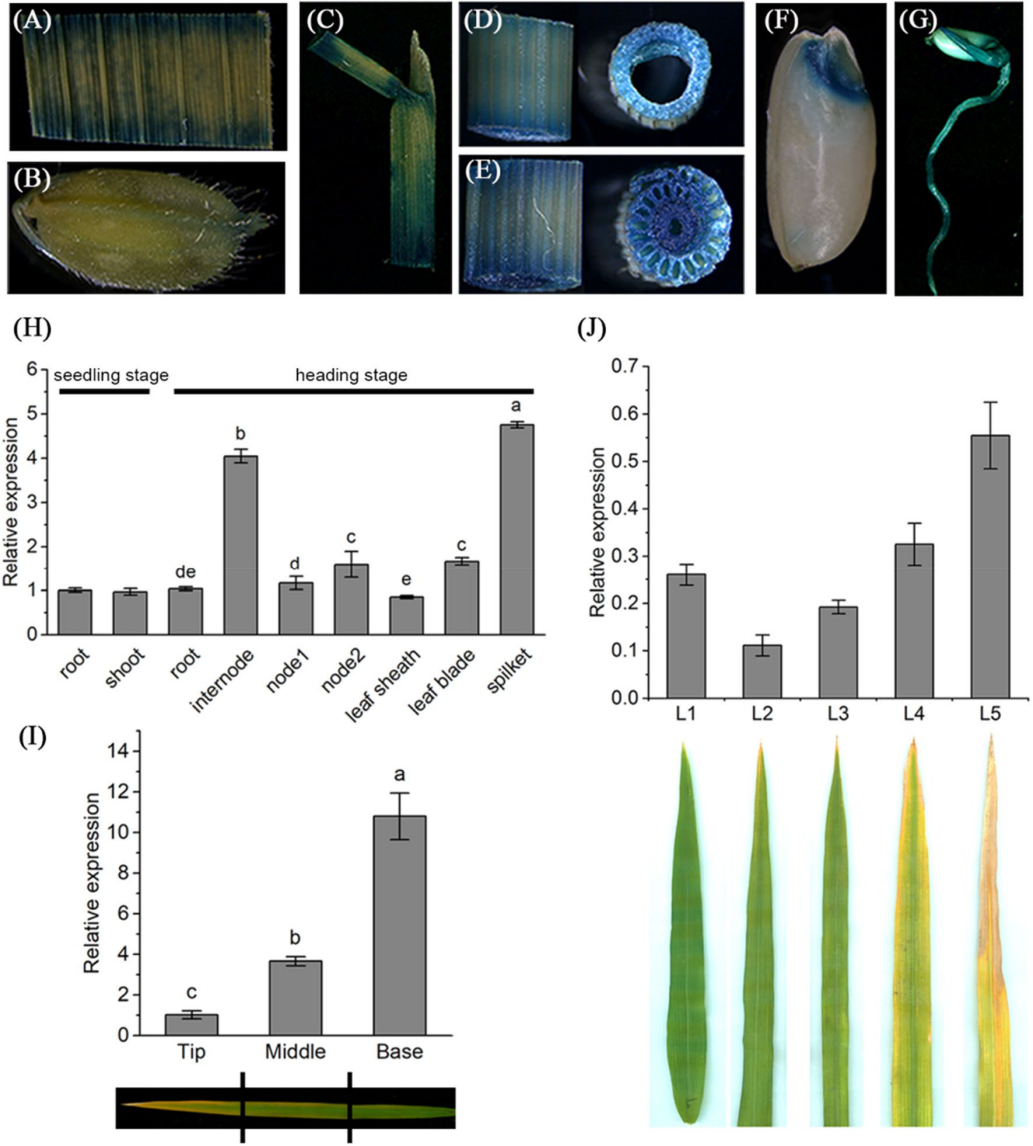
Expression pattern of *YL3*. **a**–**g**. Histochemical GUS staining of leaf (**a**), spikelet (**b**), leaf sheath (**c**), internode (**d**), node (**e**), embryo (**f**), coleoptile and radicle (**g**); **h** Expression levels of *YL3* in various organs at the seedling and heading stages (means ± SD, *n* = 3). At the seedling stage, Student’s *t* test was performed; at the heading stage, different lowercase letters above the bars indicate a statistical difference at *P* ≤ 0.05 by one-way ANOVA and Duncan’s test; **i** Expression levels of *YL3* in senescent leaves of WT (means ± SD, *n* = 3); **j** Expression of *YL3* at different growth stages. L1, flag leaf; L2, top second leaf; L3, top third leaf; L4, top fourth leaf; L5, top fifth leaf. Different lowercase letters above the bars indicate a statistical difference at *P* ≤ 0.05 by one-way ANOVA and Duncan’s test

To determine the spatial and temporal expression patterns of *YL3,* we first carried out a GUS assay by transforming the *YL3*-promoter-drived construct p1381-YL3 into Nipponbare embryogenic calli. The results showed that the GUS signals were observed in all tissues of transgenic plants, including the leaves, sheaths, spikelets, internodes, nodes, embryos, coleoptiles and radicles (Fig. 5a–g). We then performed *YL3* expression analysis, consistent with the results of GUS assay, the expression of *YL3* was detected in all the organs tested by qRT-PCR analysis (Fig. 5h). In addition, the expression level of *YL3* decreased gradually from the leaf tip to the base in a naturally senescent WT leaf, suggesting that *YL3* might negatively regulate leaf senescence in a single senescent leaf (Fig. 5i). However, the expression of *YL3* was quite different in an individual plant at different stages compared to that in a single leaf blade. In fact, the expression of *YL3* decreased from the bottom to the top leaves (leaf 5 to leaf 1) except the flag leaf (leaf 1) with the development and senescence of an individual plant (Fig. 5j). This result indicated that NAC109 might simultaneously regulate plant growth and development besides leaf senescence at the reproductive growth stage.

### *YL3* encodes a transcriptional factor OsNAC109

**Fig. 6:**
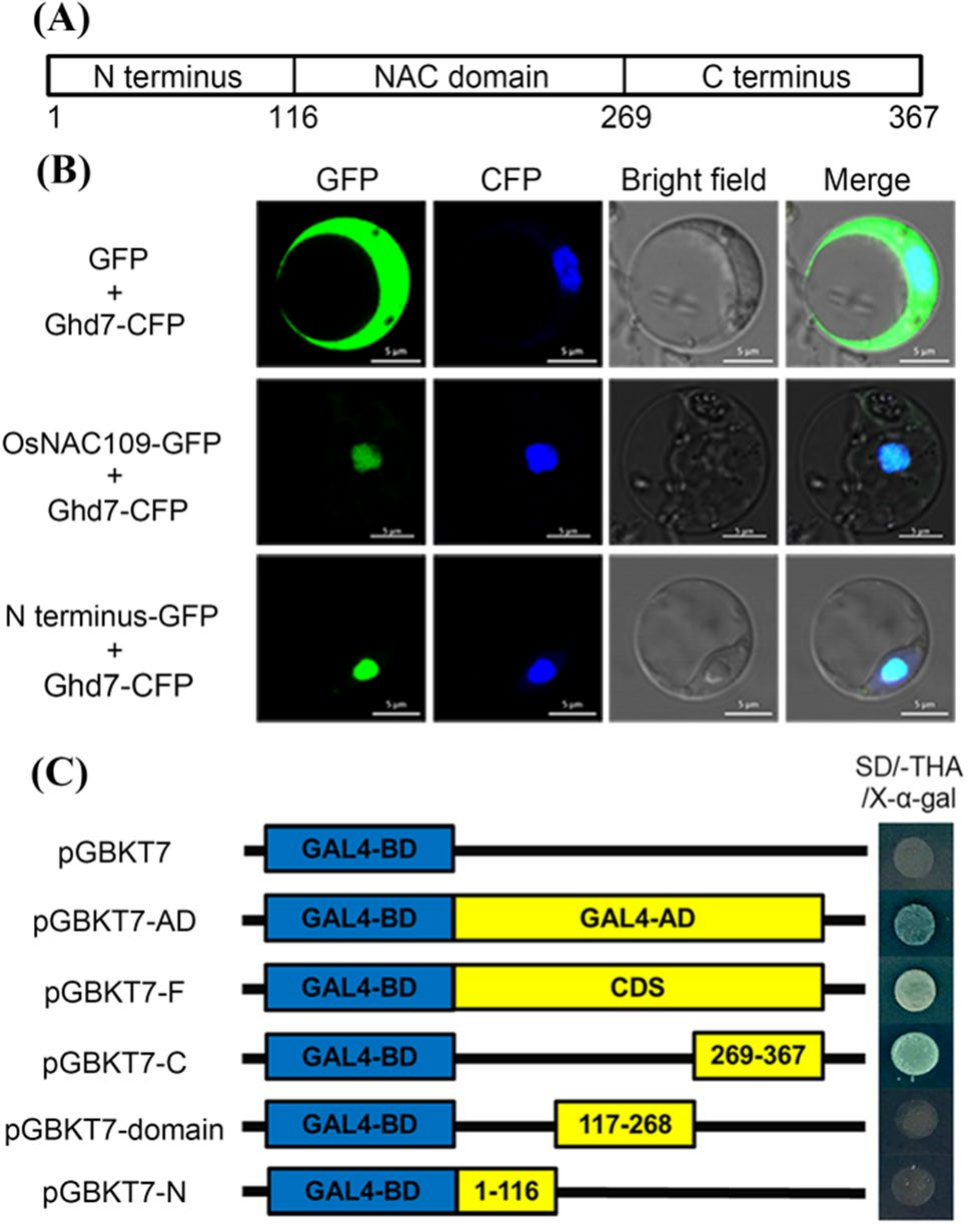
Subcellular localization and transcriptional activation assay of OsNAC109. **a** Structure of OsNAC109 including N-terminus (1–116), NAC domain (117–268) and C-terminus (269–367); **b** Subcelluar localization of OsNAC109; **c** Transcriptional activation assay of OsNAC109. *GAL4-BD* GAL4 DNA-binding domain, *GAL4-AD* GAL4 activation domain, *CDS* coding sequence of *OsNAC109*

*YL3* encodes a putative NAC transcription factor OsNAC109 with a typical NAC domain containing five subdomains, and is closely related to the Arabidopsis AtNAC57 (Supplementary Fig. S4). To examine the subcellular localization of OsNAC109, the construct PAN-F was transiently expressed together with the nuclear localization marker pCFP-Ghd7 in rice protoplasts derived from WT (Gao et al. 2014). The co-localized fluorescent signals from GFP and CFP in nuclear region suggested that OsNAC109 is a nucleus-localized protein. To determine the nuclear localization sequence which is predicted to localize in the N-terminus of OsNAC109 by the cNLS mapper (http://nls-mapper.iab.keio.ac.jp), we cotransformed the vector PAN-N and pCFP-Ghd7 into the rice protoplasts derived from WT. The results showed that the GFP and CFP signals merged perfectly in the nucleus, confirming that the nuclear localization sequence was indeed present in the N-terminus of OsNAC109 (Fig. 6b).

To further investigate whether OsNAC109 possessed the transcriptional activity, the constructs pGBKT7-F, pGBKT7-N, pGBKT7-domain, pGBKT7-C, and pGBKT7-AD, were transformed into the yeast strain Y2HGold. The results indicated that the transformants carrying pGBKT7-AD, pGBKT7-F and pGBKT7-C were able to grow whereas the transformants carrying pGBKT7 (negative control), pGBKT7-domain, and pGBKT7-N did not grow on SD/-Trp/-His/-Ade medium (Fig. 6c). These results indicated that OsNAC109 possessed a transcriptional activator localized to the C-terminus.

### OsNAC109 directly regulates transcription of senescence and hormone-associated genes by binding to the CNTCSSNNSCAVG element

**Fig. 7:**
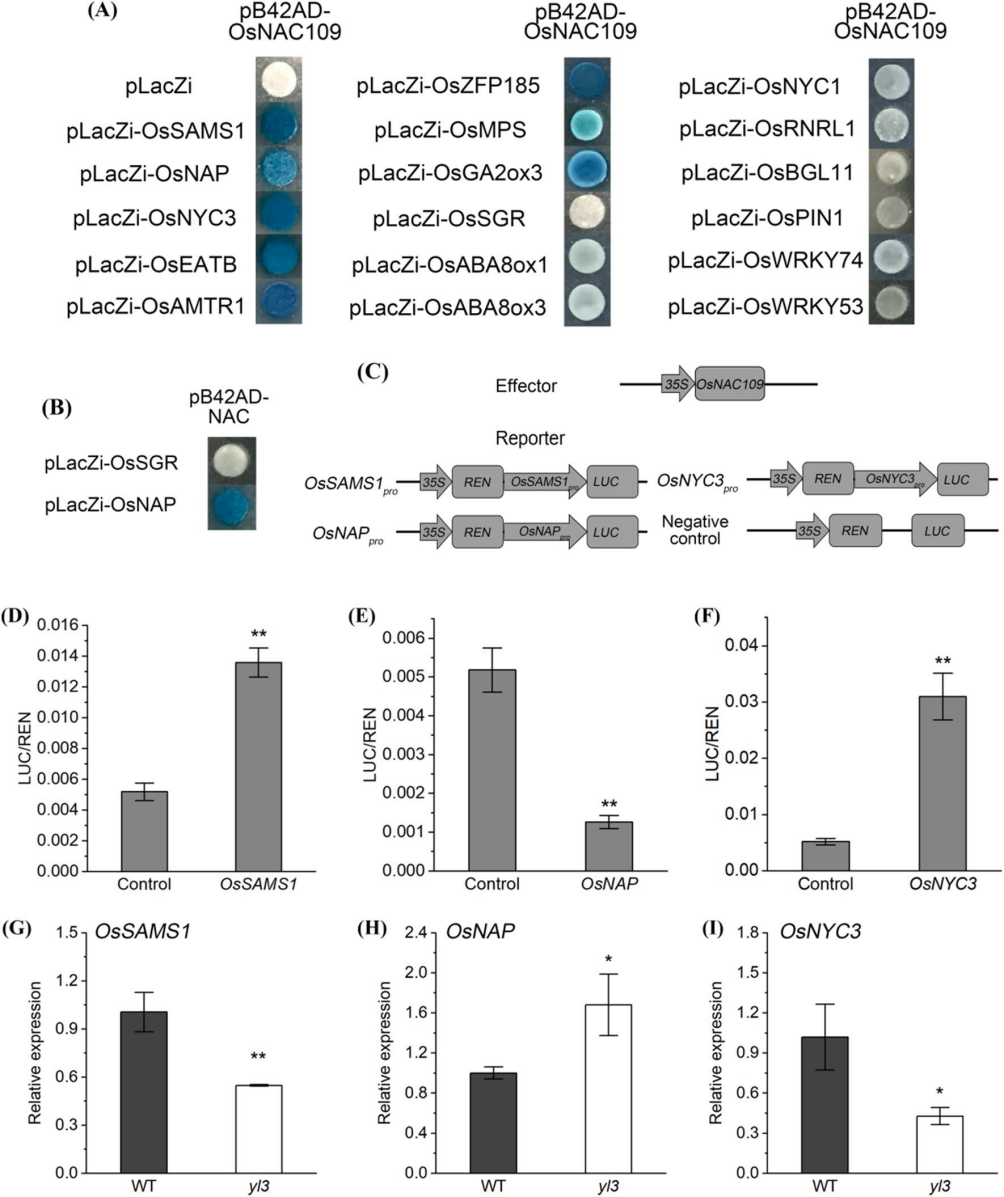
Yeast one-hybrid assay and *OsSAMS1*, *OsNAP* and *OsNYC3* expression analysis. **a** Yeast one-hybrid assay of OsNAC109. The empty negative control pLacZi was co-transformed with effectors; **b** Yeast one-hybrid assay of the NAC domain of OsNAC109; **c** Diagram of the effector and reporter constructs used in **d**–**f**; *REN* indicates *Renilla* luciferase gene, and *LUC* indicates firefly luciferase gene; **d**–**f** Dual luciferase reporter assay of *OsSAMS1* (**d**), *OsNAP* (**e**), and *OsNYC3* (**f**), means ± SD, *n* = 3; **P < 0.01 by Student’s *t* test; **g**–**i** Expression levels of *OsSAMS1* (**g**), *OsNAP* (**h**), and *OsNYC3* (**i**) in WT and *yl3* at the seedling stage, means ± SD, *n* = 3, *P < 0.05, and **P < 0.01 by Student’s *t* test

To understand the regulatory network behind OsNAC109-mediated growth-arrest and senescence, we performed the transcriptome analysis to identify differentially expressed genes (DEGs) between WT and *yl3* at the tillering stage. The results showed that a total of 2200 DEGs were identified between *yl3* and WT (Supplementary Table S3). Considering the senescent phenotype of *yl*3, we focused on SAGs, chlorophyll metabolism-associated and hormone metabolism-related genes, and 13 DEGs were selected for further validation by yeast one-hybrid assay (Supplementary Data S1, total). In addition, *OsRNRL1*, *OsSGR*, *OsNYC1* and *OsNYC3* that were not differentially expressed in transcriptome analysis but are likely target genes of OsNAC109 according to the PlantTFDB database (http://planttfdb.cbi.pku.edu.cn/) and previous studies (Yoo et al. 2009; Liang et al. 2014; Sakuraba et al. 2015; Mao et al. 2017) were selected for yeast one-hybrid assay (Supplementary Table S3). The results indicated that OsNAC109 could directly regulate the expression of *OsSAMS1*, *OsNAP, OsNYC3, OsEATB*, *OsAMTR1*, *OsZFP185*, *OsMPS* and *OsGA2ox3* (Fig. 7a), indicating OsNAC109 modulated *yl3* growth and senescence by targeting a set of SAGs, chlorophyll metabolism-associated and hormone metabolism-related genes. The NAC domain of OsNAC109 possessing the DNA-binding activity was also verified with evidence that the activation of *LacZ* reporter was predominant in the transformants carrying pB42AD-NAC and placZi-OsNAP, whereas the *LacZ* reporter was deactivated completely in the transformants carrying pB42AD-NAC and pLacZ-OsSGR (Fig. 7b).

It has been shown that NAC TF recognizes specific NACRS such as the CNTNNNNNNNANG element to activate the expression of downstream genes (Supplementary Fig. S5a; O’Malley et al. 2016). To determine the specific NACRS that could be bind by OsNAC109 in rice, we performed electrophoretic mobility shift assay (EMSA) on 10 potential sequences in the promoters of selected genes including *OsNAP*, *OsNYC3*, *OsSAMS1*, *OsEATB*, *OsAMTR1* and *OsZFP185* (Supplementary Fig. S5b). The results showed that the GST-OsNAC109 fusion protein could bind to the biotin-labeled DNA fragments P1, C1, B1, A1 and Z1 (Supplementary Fig. S5c), which all possess a highly conserved CNTCSSNNSCAVG sequence (Supplementary Fig. S5d). To further validate whether OsNAC109 bind to CNTCSSNNSCAVG, we then generated three P1 variants, M1, M2 and M3 (Supplementary Fig. S5e). The results showed that the binding ability of OsNAC109 to P1 fragments was gradually decreased with the increasing amounts of unlabeled P1. The binding ability of OsNAC109 to labelled P1 was apparently inhibited by M2, but almost unaffected by the unlabeled M1 and M3 (Supplementary Fig. S45f). Although GST-OsNAC109 was able to bind to the promoter of *OsSAMS1*, the NACRS for *OsSAMS1* has yet to be determined. Taken together, our results demonstrated that OsNAC109 bind to the conserved CNTCSSNNSCAVG element in the promoters.

It has been reported that *OsSAMS1*, *OsNAP* and *OsNYC3* regulate leaf senescence in rice. To test whether OsNAC109 associated with the leaf senescence in *yl3*, we carried out a dual-luciferase reporter assay using rice protoplasts (Fig. 7c). As shown in Fig. 7d–f, the LUC activities under the control of the promoters of *OsSAMS1*, *OsNAP* and *OsNYC3* were approximately 2.62-, 0.24- and 5.98-fold compared with the control. In addition, at the seedling stage, the relative expression of *OsSAMS1* and *OsNYC3* were significantly down-regulated in *yl3* while the expression of *OsNAP* was apparently up-regulated in *yl3* compared with WT (Fig. 7g–i), consistent with the dual-luciferase reporter assay. Taken together, we concluded that OsNAC109-mediated *yl3* leaf senescence was associated with the up-regulation of *OsSAMS1* and *OsNYC3*, and down-regulation of *OsNAP*.

### OsNAC109 regulates plant hormone biosynthesis in rice

**Fig. 8:**
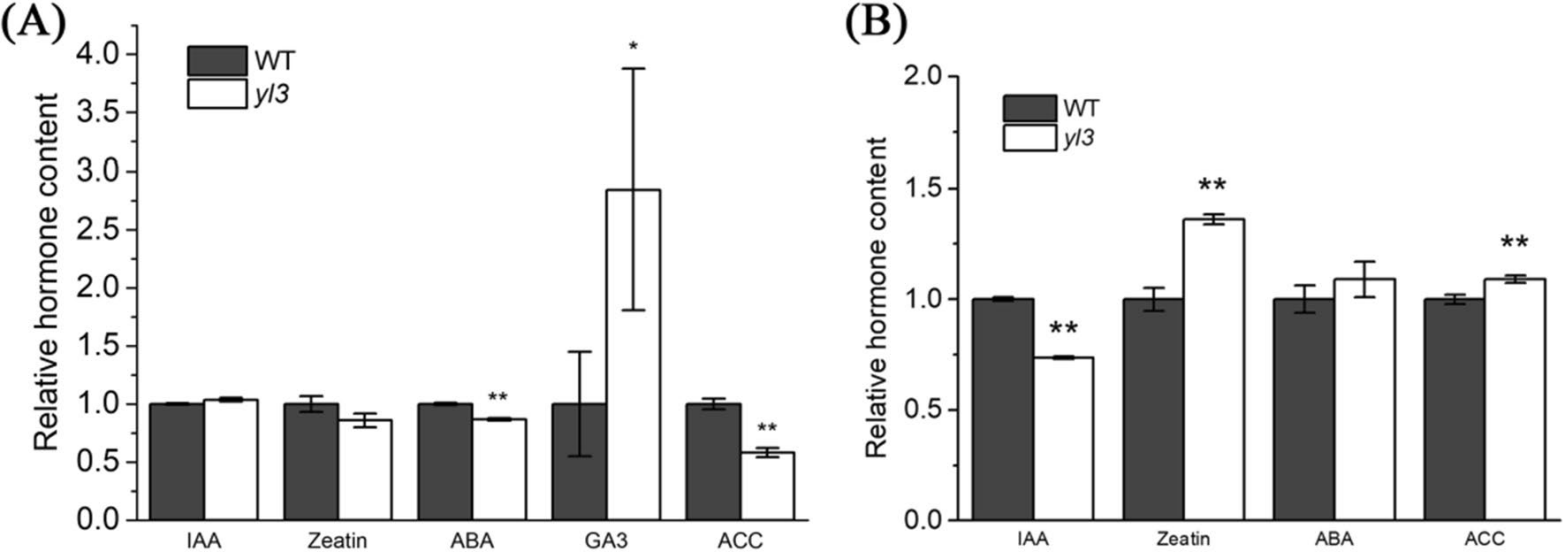
Relative hormone content in WT and *yl3* at the seedling and heading stages. **a** Hormone contents of WT and *yl3* at the seedling stage; **b** Hormone contents of WT and *yl3* at the heading stage. *IAA* indole-3-acetic acid, *ABA* abscisic acid, *GA3* gibberellin A3, *ACC* 1-aminocyclopropane-1-carboxylic acid. Values are means ± SD, *n* = 3, *P < 0.05, and **P < 0.01 by Student’s *t* test

We have shown above that OsNAC109 interacted with *OsSAMS1*, *OsNAP*, and *OsGA2ox3*. Among them, OsSAMS1 catalyzes the synthesis of the ethylene precursor S-adenosyl-l-methionine (SAM); OsNAP is a NAC transcriptional factor in ABA biosynthesis; and OsGA2ox3 is responsible for GA biosynthesis (Lo et al. 2008; Chen et al. 2013; Liang et al. 2014). To verify whether the mutation of *OsNAC109* affected the hormone level in *yl3,* we detected the level of endogenous plant hormones in the leaves of WT and *yl3* at the seedling stage. The results showed that the contents of ABA and 1-aminocyclopropane-1-carboxylic acid (ACC), an indicator of ethylene, in *yl3* were significantly lower than those of WT. In contrast, the level of GA3 increased significantly in *yl3* while the levels of IAA and zeatin were similar between *yl3* and WT (Fig. 8a). In addition, ABA metabolic gene expression revealed that ABA degradation genes such as *OsABAox1*, *OsABAox2* and *OsABAox3* were up-regulated, while the ABA biosynthesis gene, *OsNCED1*, was apparently down-regulated in *yl3* (Supplementary Fig. S6a-f). Furthermore, the transcription levels of most GA biosynthesis genes tested were up-regulated in the mutant (Supplementary Fig. S6g-l). It was noticed that the expressions of ABA and GA-associated genes among the knockout lines were not consistent similar to their phenotypes possibly due to the target sites (Supplementary Fig. S7a-b). Furthermore, we detected the contents of endogenous plant hormones in the leaves of WT and *yl3* at the heading stage when senescence was initiated, and the results showed that the zeatin and ACC levels were increased, the content of IAA was decreased, whereas the ABA level was similar in *yl3* compared to WT (Fig. 8b). Taken together, the results indicated that the mutation of *OsNAC109* altered the expression of hormone metabolism genes and hormone levels at the seedling stage, and affected hormone levels at the heading stage when leaf senescence was initiated in *yl3*.

## Discussion

In the present study, we isolated and characterized a novel *yellow leaf 3* mutant from an EMS-induced Zhongjian 100 mutant bank. As a complex and highly programed process, leaf senescence generally manifests yellowing of leaves phenotypically. Like many other senescence mutants, *yl3* senescent leaves undergo a series of physiological/biochemical changes such as degradation of chlorophyll, lipid, protein and nucleic acids, enhancement of MDA content, decreased activities of ROS scavenging enzymes such as CAT, POD and SOD, and the accumulation of ROS (Wittenbach 1977; Hua and Wang 2003). ROS such as superoxide, hydrogen peroxide (H_2_O_2_) and superoxide radical (O_2_^−^) are viewed as senescence-associated toxic molecules that lead to lipid peroxidation, cellular damage and cell death, and simultaneously impact gene expression as signals (Foyer and Noctor 2005). In plants, excessive amount of ROS is eliminated by ROS scavenging enzymes, however, previous studies showed that the enzymatic activity changes are inconsistent and controversial. For example, the SOD activity is apparently higher while the CAT and POD activities were lower in rice early senescence mutant *es4* compared with the wild type (Wang et al. 2019). In rice *wls5*, a weak and leaf premature mutant, the POD activity was significantly higher and the CAT activity was apparently lower than those of wild type (Zhao et al. 2019). It has been shown that the SOD and CAT activities were significantly reduced, but POD unaltered in *psl85* compared to the wild type (He et al. 2018). Here, we also found that the activities of both SOD and CAT were greatly decreased in the uppermost leaves of *yl3* compared with WT, while the activity of POD was similar between *yl3* and WT (Fig. 2h–j). We speculated that the reduced activities of SOD and CAT in *yl3* were likely responsible for the over-accumulation of ROS, which resulted in the onset and acceleration of senescence. Although the CAT activity decreased, the expression of *CATA* and *CATB* was obviously increased. This phenomenon was likely caused by a feedback for the reduction of CAT activity, or different detection stages as the activity of CAT was detected at the tillering stage but the expression of *CATA* and *CATC* was measured at the seedling stage. The impacted expression of ROS-associated genes in *yl3* also indicated that OsNAC109 was likely involved in the regulation of ROS-associated genes. Additionally, premature leaf senescence is generally accompanied by retarded growth and development. In Arabidopsis, the *mosaic death* 1 (*mod1*) and the corresponding *RPI2-*knockout lines are chlorotic and semidwarf (Mou et al. 2000; Xiong et al. 2009). In rice, *wls5* exhibits early leaf senescence and weak growth (Zhao et al. 2019). Similarly, besides of early senescence phenotype, *yl3* also shows dwarfism owing to the shortened cell length in each internode (Fig. 1h–l). Therefore, *yl3* is a typical premature senescent mutant manifested by yellowish leaves together with arrested growth and development.

We isolated the causal gene *YL3* responsible for leaf premature senescence and arrested-growth of *yl3*, and the amino acid sequence alignment suggested that *YL3* was a novel gene encoding a transcriptional factor OsNAC109 harboring a typical conserved NAC domain. It is noted that the *yl3* trait did not fit to the normal 3:1 segregation ratio due to unknown reasons, however, the functional complementation by *YL3* supported a single gene control of the *yl3* phenotype. *YL3* was widely expressed like many other senescence-associated genes and NAC transcriptional factors in previous studies (Huang et al. 2016; Shim et al. 2018; Wang et al. 2019, 2020; Liu et al. 2020), and this expression pattern also explained that OsNAC109 was able to regulate various developmental processes, as demonstrated for the NAC transcription factors described previously (Souer et al. 1996; Aida et al. 1997). Interestingly, although NAC transcriptional factors mainly localize to the nuclei, some NACs may possess extra transmembrane domains in the C-terminus (Seo and Park 2010; Kim et al. 2012; Liu et al. 2020; Sakuraba et al. 2020). In our present study, we demonstrated that OsNAC109 possessed a nuclear localization sequence in the N-terminus, in contrast, the C-terminus did not contain a transmembrane domain instead it acted as a transcriptional activator likely associated with plant growth and senescence (Fig. 6b, c). According to the previous studies, some NAC factors, NTL4 (Lee et al. 2012), OsNAC5 (Sperotto et al. 2009), OsNAC6 (Nakashima et al. 2007), ORE1 (Kim et al. 2014), OsNAC2 (Mao et al. 2017), ONAC106 (Sakuraba et al. 2015) and OsNAP (Liang et al. 2014) are involved in aging process. For instance, ONAC106 acts as a leaf senescence inhibitor by directly mediating the expression of SAGs such as *OsSGR* and *OsNYC1* (Sakuraba et al. 2015). *OsNAP* directly controls the expression of SAGs, *OsSGR*, *OsNYC1*, *OsNYC3*, *OsRCCR1* and *OsI57* as well as represses ABA biosynthesis, acting as an aging inducer (Liang et al. 2014). OsNAC2 mediates leaf senescence by regulating the expression of SAGs, and simultaneously controls ABA metabolism (Mao et al. 2017; Shen et al. 2017). Additionally, NAC transcriptional factors regulate various biological processes via different hormone pathways in plants. OsNAC2 is also involved in different hormone pathways, such as auxin, cytokinin and gibberellic acid, to regulate plant growth and development (Chen et al. 2015; Mao et al. 2020). Furthermore, ONAC066 regulates disease resistance by suppressing the ABA signaling pathway in rice (Liu et al. 2018). In Kiwifruit, NAC factors participate in the cross-talk between methyl jasmonate and ethylene (Wu et al. 2020). Arabidopsis NAC transcription factor JUB1 regulates GA/BR metabolism and signaling (Shahnejat-Bushehri et al. 2016). Here, it is found that the mutation of *OsNAC109* triggered a huge transcription alteration of genes associated with senescence, photosynthesis and hormone metabolism (Fig. 3a, b; Supplementary Fig. S6). OsNAC109 targeted a set of SAGs, chlorophyll and hormone metabolism-related genes including *OsSAMS1*, *OsNAP, OsNYC3, OsEATB*, *OsAMTR1*, *OsZFP185*, *OsMPS* and *OsGA2ox3* (Fig. 7a)*.* It has been shown that *OsSAMS1*, encoding S-ADENOSYL-l-METHIONINE SYNTHETASE 1, is involved in ethylene biosynthesis (Chen et al. 2013) and *OsNYC3* plays an important role in chlorophyll degradation (Morita et al. 2009). In the present studies, we found that OsNAC109 promoted the expression of *OsSAMS1* and *OsNYC3*, while repressed the expression of *OsNAP* (Fig. 7c–i). It was noticed that *OsNYC3*, as a target gene enhanced by OsNAP, was up-regulated whereas the *OsNAP* expression was suppressed in *yl3*. The observation indicated that *OsNYC3* was impacted mainly by the mutation of *OsNAC109* rather than the modest reduction expression of *OsNAP* in *yl3*, and. OsNAC109 was probably associated with leaf senescence in rice. Furthermore, the identification of NACRS in transcriptional factors is helpful to reveal the molecular mechanism at the transcriptional level. Previous studies showed that most NAC transcriptional factors recognize and bind the sequence containing highly conserved CNT and ANG elements in Arabidopsis (O’Malley et al. 2016). However, the NACRS of NAC transcriptional factors are largely unknown in rice. Here, we identified a highly conserved core binding site of OsNAC109, CNTCSSNNSCAVG from a set of hormone/senescence-associated genes except *OsSAMS1* whose NACRS has yet to be clarified (Supplementary Fig. S5), and this would be helpful for the identification of potential target genes for further understanding OsNAC109-mediated mechanism regulating plant senescence, growth and development.

Phytohormones play a key role in plant development including growth, reproduction and senescence. ABA is shown to participate in leaf senescence in the plant kingdom (Becker and Apel 1993). Previous studies suggested that ABA might promote plant senescence by inducing the expression of some SAGs, such as *OsNYC1* (Kusaba et al. 2007), *SGR* (Park et al. 2007), *PPH* (Schelbert et al. 2009). Overexpression of *OsNAC2* leads to leaf early senescence and increases ABA level, whereas the knockdown lines show delayed-leaf senescence with reduced ABA level in rice (Mao et al. 2017). Notably, Overexpression of *OsNAP* significantly accelerates leaf senescence with decreased ABA content, whereas knockdown of *OsNAP* produces delayed leaf senescence with overproduction of ABA (Liang et al. 2014). In this study, although *yl3* showed significantly decreased content of ABA at the seedling stage, while its content recovered to the WT level at the heading stage when senescence was initiated in *yl3*, indicating the enhanced ABA level at the seedling stage was unlikely associated with leaf senescence. Ethylene affects plant development at the vegetative growth stage, and is well-known to be an endogenous regulator of plant leaf senescence and fruit ripening. It has been showed that ethylene promotes plant senescence (Wang and Woodson 1989; Savin et al. 1995; Huang et al. 2007; Chen et al. 2013). We also noticed that the content of ACC was increased significantly at the heading stage of *yl3* when leaf senescence had initiated although its content decreased at the seedling stage of *yl3*. The decline of ethylene production resulted from the suppression of carnation ACC oxidase during flower senescence could enhance the longevity of flowers (Savin et al. 1995). Overexpression of *OsFBK12* and knockdown of *OsSAMS1* cause decreased ethylene production and delayed leaf senescence, in contrast, overexpression of *OsSAMS1* and knockdown of *OsFBK12* lead to early leaf senescence with an increased ethylene level (Chen et al. 2013). Here, *yl3* exhibited markedly decreased content of ethylene because of down-regulation of ethylene precursor synthetase gene *OsSAMS1* at the seedling stage, in contrast, the ethylene was obviously higher at the heading stage of *yl3* than that of WT, indicating that ethylene was likely involved in leaf senescence at the reproductive growth stage of *yl3*. In fact, ethylene-mediated senescence is complex and treatments by different phytohormones results in ethylene-responsive-like phenotypes, implying a complicated hormone cross-talk in rice (Agarwal et al. 2012; Lee and Yoon 2018). In addition, GA was mainly responsible for stem elongation, seed germination and leaf expansion (Monna et al. 2002; Fu and Harberd 2003; Kaneko et al. 2003; Xie et al. 2006). In rice, overexpression of *OsNAC2* inhibits plant height by directly mediating the key components of GA pathway (Chen et al. 2015). In the present study, *yl3* exhibited dwarfism and arrested-growth with apparently increased GA content and up-regulation of at least six GA biosynthetic genes at the seedling stage (Supplementary Fig. S6g-l). Therefore, OsNAC109- regulated plant growth, development and senescence was probably associated with multiple hormone metabolism pathways covering ABA, GA and ethylene as manifested by *OsNAP*, *OsGA2ox3* and *OsSAMS1*. Furthermore, it has been reported *OsEATB*, *OsMPS*, *OsZFP185* are involved in plant growth and development associated with hormone responses to biotic/abiotic stresses (Qi et al. 2011; Schmidt et al. 2013; Zhang et al. 2016). It was noticed that OsNAC109 was able to bind to the promoters of *OsEATB*, *OsMPS*, and *OsZFP185* in the present study but further studies are required to clarify their roles in association with senescence, growth and development in rice. Taken together, we speculated that OsNAC109 is involved probably in regulation of leaf senescence and growth via the cross-talk of multiple hormone pathways.

In summary, we isolated and characterized a rice *yellow leaf 3* mutant displaying premature leaf senescence and retarded growth due to a single base nucleotide substitution of a NAC transcription factor gene *OsNAC109*. OsNAC109 could specifically recognize a highly conserved DNA cis-element CNTCSSNNSCAVG to modulate the leaf senescence, plant growth and development by altering the expression of a series of hormone- and senescence-associated genes.

## Supplementary Information

Below is the link to the electronic supplementary material.

Supplementary file1 (DOCX 1904 KB)

Supplementary file2 (XLSX 7431 KB)
