# Supplementary material for: OsNAC109 regulates senescence, growth and development by altering the expression of senescence- and phytohormone-associated genes in rice
Source: Plant Mol Biol. 2021 Feb 4;105(6):637–54. doi: 10.1007/s11103-021-01118-y (PMC7985107; doi:10.1007/s11103-021-01118-y)
Supplement: Supplementary file 1 — Supplementary file1 (DOCX 1904 KB) [file 11103_2021_1118_MOESM1_ESM.docx]

**
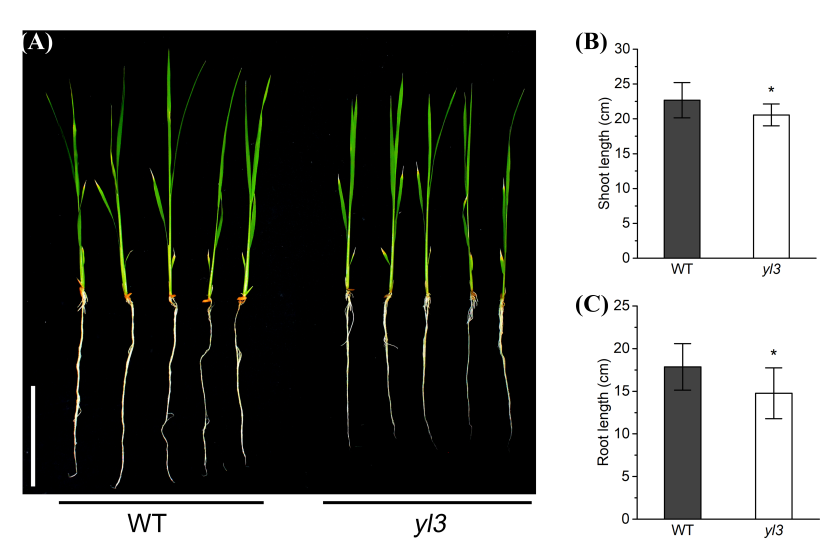
**

**Figure. S1** Comparison of shoots and roots between *yl3* and WT

**a**. The phenotype of two-week hydroponically cultured seedlings from WT and *yl3*; **b**. The shoot length of two-week seedlings from WT and *yl3*; c. The root length of two-week seedlings from WT and *yl3*. Values are means ± SD (*n* = 10). * indicates significance at *P* < 0.05 by Student’s *t*-test.





**Figure. S2** Relative expression analysis of ROS-related genes in *yl3* and WT at 3-week old seedlings. Data are means± SD, *n*=3. ** indicates significance at *P<*0.01 by Student’s *t*-test.


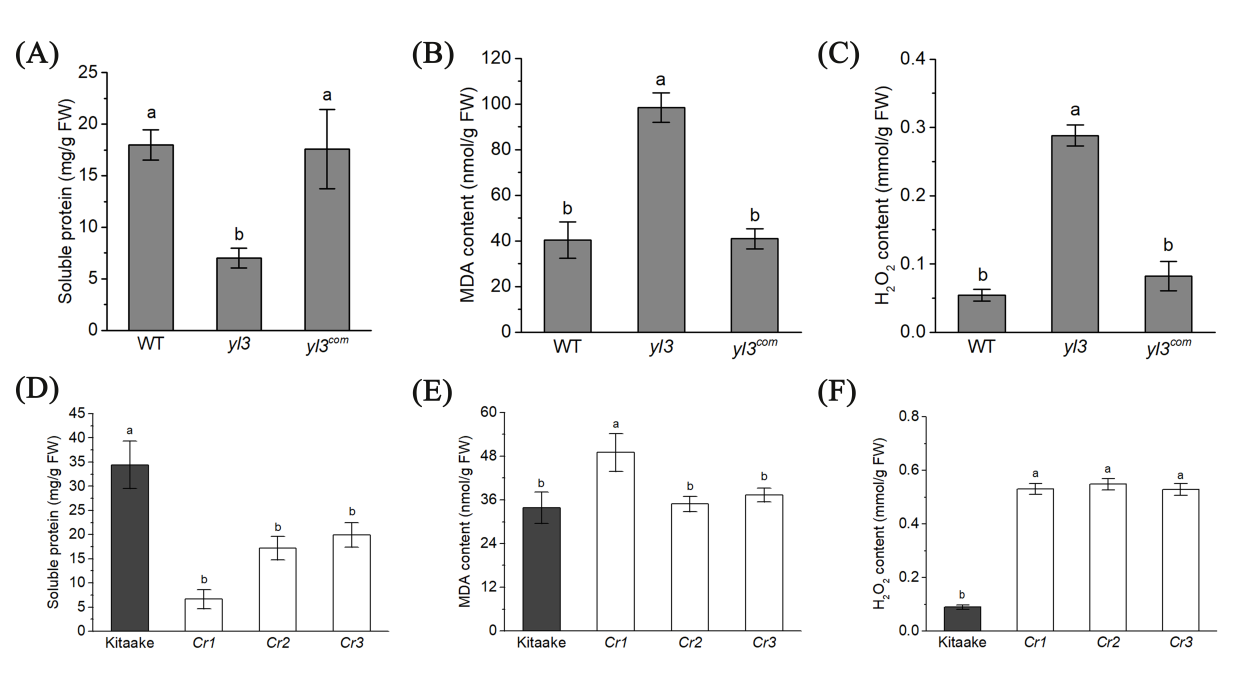


**Figure. S3** The detection of physiological parameters related to senescence in the complementation lines and knockout lines

**a**, **d** The content of total soluble protein in the complementation lines (**a**) and knockout lines (**d**) ; **b**, **e** The content of MDA in the complementation lines (**b**) and knockout lines (**e**) ; **c**, **f** The content of H_2_O_2_ in the complementation lines (**c**) and knockout lines (**f**). Values are means ± SD (*n* = 3). Different lowercase letters above the bars indicate a statistical difference at *P* ≤ 0.05 by one-way ANOVA and Duncan’s test.


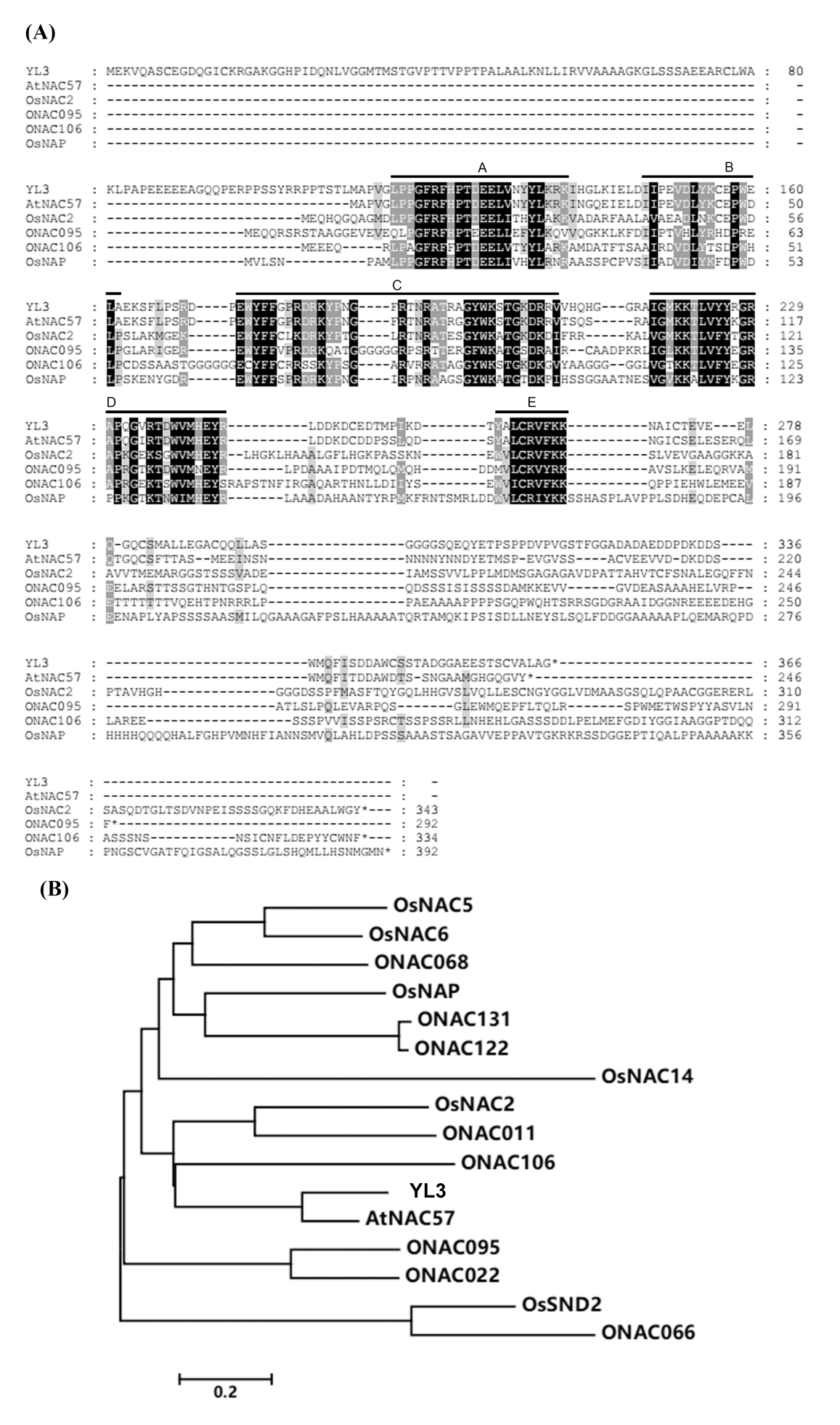


**Figure. S4** Sequence comparison and phylogenetic analysis

**a** Amino acid sequence alignment among YL3, OsNAP, OsNAC2, ONAC095, ONAC106, and AtNAC57. Conserved amino acids are indicated with black and gray background. A-E letters represent five subdomains in the NAC domain; **b** Phylogenetic tree analysis of YL3. Phylogenetic tree was constructed by neighbor-joining method using the MEGA program. Bootstrap values from 100 replicates are indicated at each node.


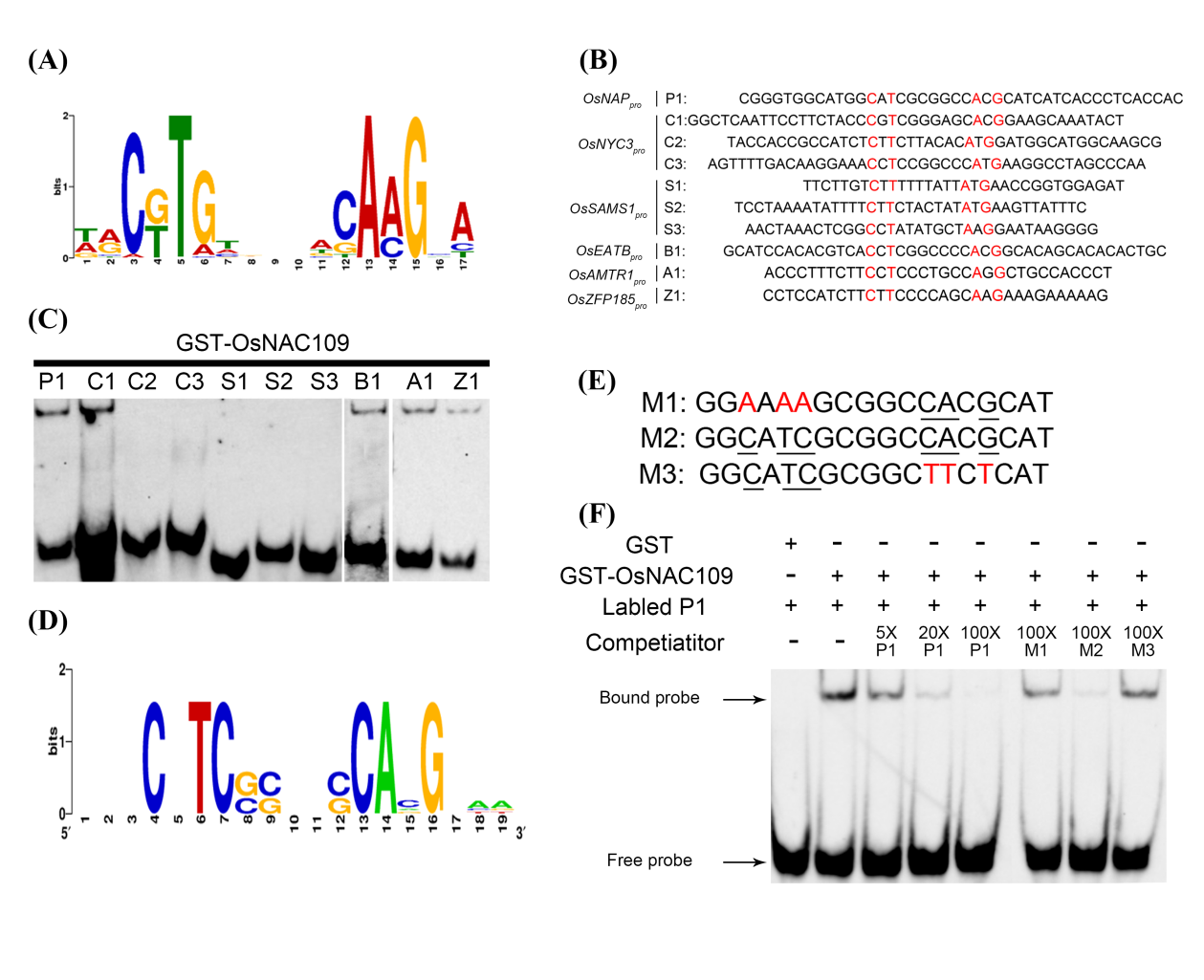


**Figure. S5** Identification of OsNAC109 binding sites

**a**. The NAC recognization sequence (NACRS) of AtNAC57; **b**. Selected fragments from the target promoters of OsNAC109; **c**. EMSA analysis shows that GST-OsNAC109 fusion protein could bind to the biotin-labeled DNA fragments P1, C1, B1, A1 and Z1; **d**. A highly conserved sequence in the DNA fragments bound by OsNAC109. **e**. Three variants of P1 sequence. The conserved nucleotides are underlined and red letters indicate variances; **f**. Binding competition of labeled P1 to OsNAC109 among the unlabeled fragments of P1, M1, M2, and M3 under multiple dilutions of labeled P1 probe.

**
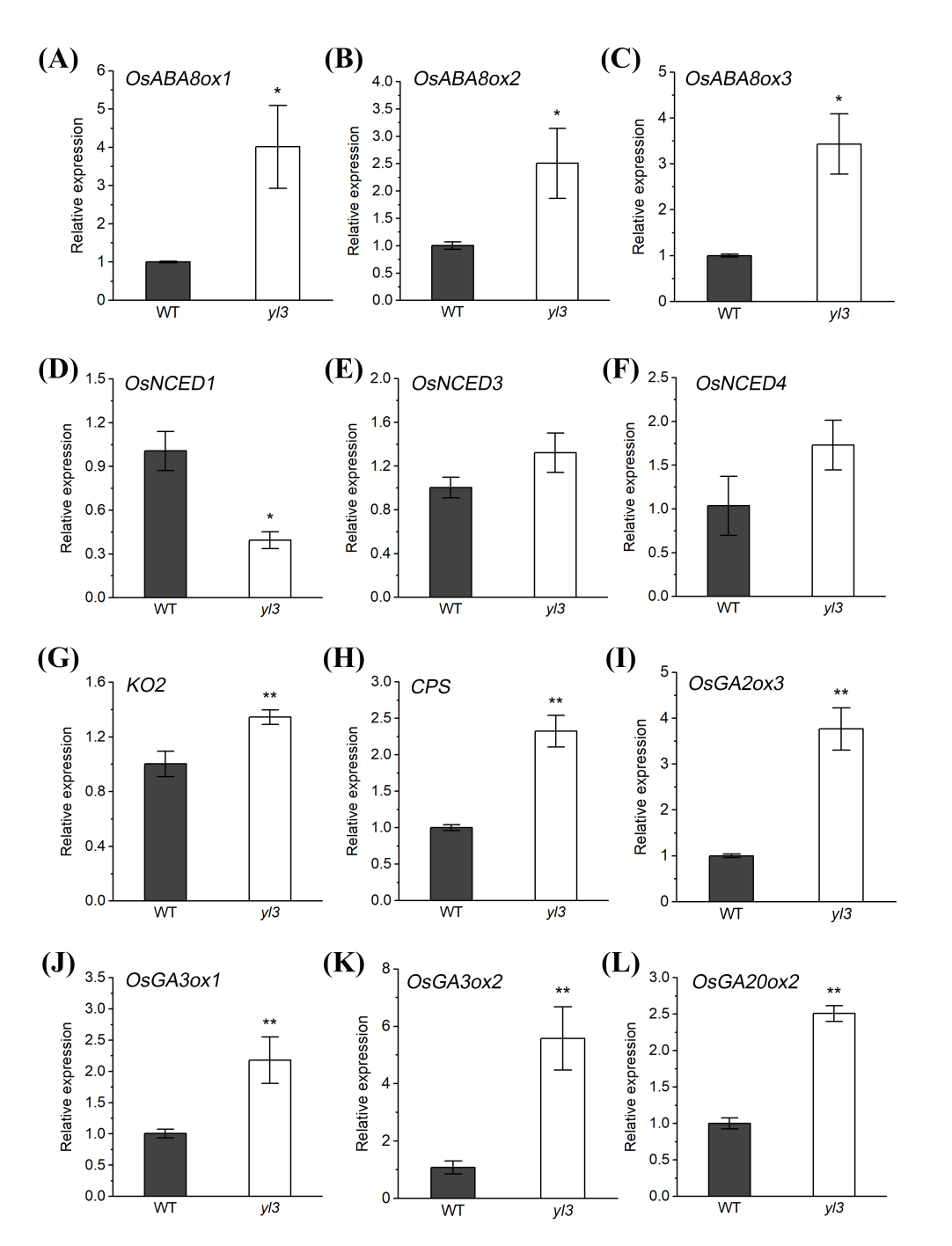
**

**Figure. S6** The expression of ABA and GA metabolism genes in leaves of WT and *yl3* seedlings

**a-c**. Expression of ABA inactivation genes; **d**-**f** Expression of ABA biosynthetic genes; **g**-**l**. Expression of GA biosynthetic genes. Values are means ± SD, *n*=3; * indicates significance at *P<*0.05, ** indicates significance at *P<*0.01 by Student’s *t*-test.


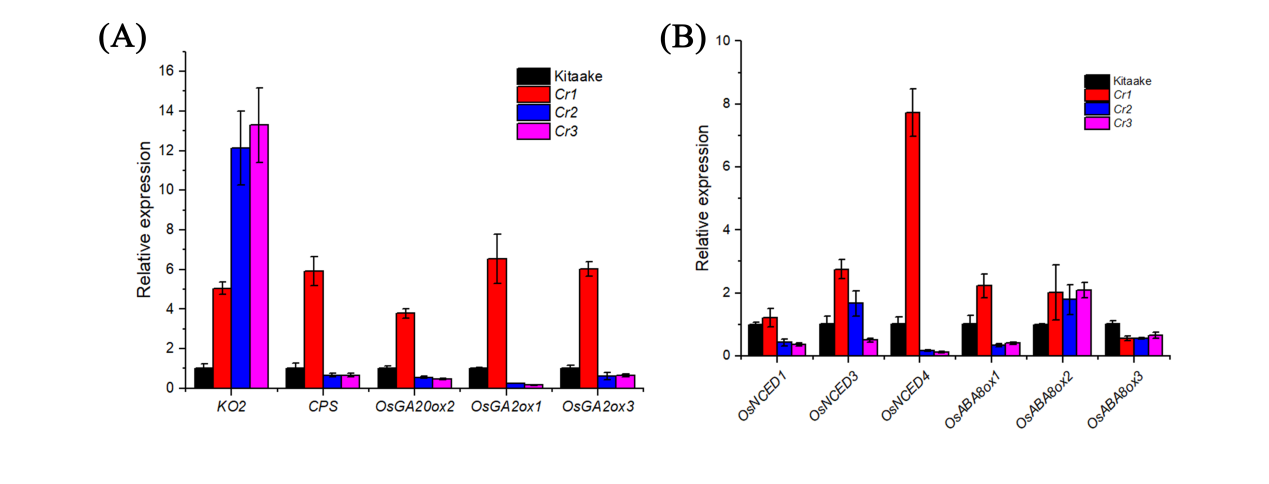


**Figure. S7** The expression of ABA and GA metabolism genes in leaves of knockout lines

**a**. Expression of GA biosynthetic genes; **b**. Expression of ABA inactivation and biosynthetic genes; Values are means ± SD, *n*=3.

**Table S1** Comparison of agronomic traits between the wild type and *yl3*

| **Trait** | **WT** | ***yl3*** |
| --- | --- | --- |
| Plant height (cm) | 101.67±2.89 | 71.67±2.08** |
| Panicle length (cm) | 26.6±0.53 | 16.93±1.20** |
| Number of productive panicles per plant | 14.67±0.58 | 15±1.73 |
| Seed setting rate (%) | 84.29%±3.17 | 48.91%±8.68** |
| 1000-grain weight (g) | 22.01±0.10 | 20.5±0.78* |

Values are means ± SD, *n*=3; * indicates significance at *P<*0.05, ** indicates significance at *P<*0.01 by Student’s *t*-test.

**Table S2** Genetic analysis of *yl3*

| Cross | F_1_ | F_2_ | | *P* (χ^2^_(3:1)_) | *P* (χ^2^_(15:1)_) |
| --- | --- | --- | --- | --- | --- |
|  |  | No. of normal plant | No. of mutant plant |  |  |
| *yl3*/Moroberekan | normal | 1587 | 245 | 1.43811E-30 | 2.22988E-36 |
| *yl3*/Zhongjian100 | normal | 473 | 83 | 4.14257E-08 | 2.82806E-17 |

**Table S3** Selected genes for yeast one-hybrid assay and their expression comparison between WT and *yl3* in transcriptome analysis

| Gene Symbol | Annotation | log2(fc) | Significance | Reference |
| --- | --- | --- | --- | --- |
| Os03g0327800 | *OsNAP* | 3.791 | yes | Liang et al., 2014 |
| Os05g0135700 | *OsSAMS1* | 0.141 | yes | Chen et al., 2013 |
| Os05g0475400 | *OsAMTR1*(*Osh36*) | 5.133 | yes | Kothari et al., 2016 |
| Os09g0457900 | *OsEATB* | 0.01 | yes | Qi et al., 2011 |
| Os02g0195600 | *OsZFP185* | 25127.37 | yes | Zhang et al., 2016 |
| Os02g0618400 | *OsMPS* | 12.40 | yes | Schmidt et al., 2013 |
| Os01g0757200 | *OsGA2ox3* | 25.74 | yes | Lo et al., 2008 |
| Os02g0703600 | *OsABA8ox1* | 0.24 | yes | Saika et al., 2007 |
| Os09g0457100 | *OsABA8ox3* | 0.20 | yes | Cai et al., 2015 |
| Os11g0592900 | *BGL11* | 0.04 | yes | Wang et al., 2013 |
| Os02g0743400 | *OsPIN1* | 6.22 | yes | Xu et al., 2005 |
| Os09g0334500 | *OsWRKY74* | 0.09 | yes | Xie et al., 2005 |
| Os05g0343400 | *OsWRKY53* | 0.25 | yes | Tian et al., 2017 |
| Os06g0168600 | *OsRNRL1* | 1.20 | no | Yoo et al., 2009 |
| Os09g0532000 | *OsSGR* | 0.723 | no | Park et al., 2007 |
| Os01g0227100 | *OsNYC1* | -0.232 | no | Kusaba et al., 2007 |
| Os06g0354700 | *OsNYC3* | -0.062 | no | Morita et al., 2009 |

Log2(fc) indicates log 2 fold change between WT and *yl3*. Yes indicates significant difference between WT and *yl3* at *P<*0.05 by Student’s *t*-test, no indicates no difference between WT and *yl3*.

**Table S4** Primers used in this study

| Primer name | Forward primer | Reverse primer |
| --- | --- | --- |
| Map-Based Cloning of *yl3* | | |
| Indel3 | GTCGTGGACCACTGGTAGTT | AGACGACTGACCGTCTCGAT |
| Indel4 | GGGCATCGTTCAGTTTGACAC | TGCTTGATTAGCCACAACCA |
| Indel5 | AGCCGGGATGTATAACTAACGA | AATTGCGTACCGAGACGGAG |
| Indel8 | CAGTAGTGCTTCCTTGAGGC | TAGCGGTGTTTTGAGGGGTG |
| Plasmid Construction | | |
| YL3-com | ggtaccCGTCCAGGATGTGCTTCGTT | cccgggTGCTAAGTGCCAACTGCAAG |
| YL3-GUS | GAATTCCCGGGGATCCTCACTGGCATTTGTACATGGCAC | GCAGGTCGACGGATCCCTCTCTCTCTCACTCACTACTAGTCTACTACTACTC |
| YL3-GFP | ACGCCTGCCGTTCGACGATTTCTAGAATGGAAAAGGTTCAAGCTTC | ACGCGCGGATCTTCCAGAGATTCCCGGGGCCGGCGAGGGCCACGCAGG |
| N-YL3-GFP | ACGCCTGCCGTTCGACGATTTCTAGAATGGAAAAGGTTCAAGCTTC | ACGCGCGGATCTTCCAGAGATTCCCGGG GAGCCCCACCGGCGCCAT |
| Transcriptional Activation Assay | | |
| YL3 | CATATGGAAAAGGTTCAAGCTTCT | GAATTCTCAGCCGGCGAGGGCCACGCAGG |
| AD | CATATGCCAAAAAAGAAGAGAAAGGTCG | GAATTCTCACTCTTTTTTTGGGTTTGGTGG |
| C-YL3 | CATATGAACGCGATCTGCACCGAGGTGGAG | GAATTCTCAGCCGGCGAGGGCCACGCAGGA |
| Domain-YL3 | CATATGCCTCCGGGGTTCCGGTTCCACCC | GAATTCTCACTTCTTGAAGACCCGGCATAA |
| N-YL3 | CATATGGAAAAGGTTCAAGCTTCTTG | GAATTCTCAGAGCCCCACCGGCGCCATCAA |
| Yeast One-Hybrid Assay and Dual-Luciferase Assay | | |
| pB42AD-YL3 | ATTATGCCTCTCCCGAATTCATGGAAAAGGTTCAAGCTTCT | GAAGTCCAAAGCTTCTCGAGTCAGCCGGCGAGGGCCACGC |
| pB42AD-NAC | GAAGTCCAAAGCTTCTCGAGTCACTTCTTGAAGACCCGGCATAAC | GAAGTCCAAAGCTTCTCGAGTCAGCCGGCGAGGGCCACGC |
| PAN-YL3  for LUC | ggaccggtcccggggATGGAAAAGGTTCAAGCTTCT | aaatgtttgaactgcaTCAGCCGGCGAGGGCCACGC |
| placZi linker | TTGAATTCGAGCTCGGTACC | GAGGTCGACAGATCCCCGGG |
| LUC linker | gtcgacggtatcgata | gctctagaactagtg |
| OsSAMS1 | CGGGCGCAGCAACCATATAAAA | TAATTAGATCCGACCGGGCACG |
| OsNYC3 | CGGCTATCGTGTCGAGATATACAA | AACTGCGAGCTCAGAACGGAGA |
| OsNAP | GCCTCAGTCCCTAAAAGGTTAAAGTT | AGATTTTTTGTCCGCTTGCGTT |
| OsSGR | TTAGGGGTAATTCGTAACCGTGG | GATTATAAGAGAGAGCGGGTTAAGTGA |
| OsEATB | GTTGGTGAACTGGGCGCAGGCC | CGTTAGCTGAAGCCGGGAGAGCG |
| OsAMTR1 | GCGACCTACTATGCTATAAAGAACATCG | CGTGTGAAGTGGTTGATGCCA |
| OsZFP185 | CTCTGATACCGTTTACACATTCTTCTTTGC | TCTCTCTCTCTTCAGGGGAAAGGT |
| OsMPS | CGCATAGATCGAACAATCATCACA | GATGCAACAACCGAGTGAGCTATC |
| OsGA2ox3 | CGAGACGGTGAGCACAACAA | ATAGGTGCATATAAGGTGAT |
| OsABA8ox1 | CACTTGTCCTTTTGCTTG | TGGCAGCTATTTATAGAT |
| OsABA8ox3 | GACAAAGCATTAGATACGGTGGTTACC | AGGGATCTTTCGAATGGTGGTG |
| OsNYC1 | TTGATTGTATGGGCCAAGTGGG | GTGTGCTCACCACTTCACCATCG |
| OsPIN1 | GAGGTGCATAGCTATTTTCT | ATGGAGAAAATATTGCGAAG |
| OsBGL11 | GGTGTTCGTCCGCGGGATGACC | GGGTTGATGAATGGACCGCGCA |
| OsWRKY74 | AGTAGTGGCAAAGGGCTGTGTG | CGCGTTGGTGGTGAGTGG |
| OsRNRL1 | GCCGCGCCACCAGCATCTATGG | GCGGATTTTGGCGTCGTGGGGT |
| OsWRKY53 | GGGTAGGGTGCACGTGTG | GGCTTAAGTAGCGGCTGC |
| qRT-PCR | | |
| Ubiquitin | CCCTCCACCTCGTCCTCAG | AGATAACAACGGAAGCATAAAAGTC |
| Osl2 | GCAGACAACAAATCGCCAAAT | TCTCCAGCAACTCTAACCAGCAT |
| OsI57 | ACCCTAAAGTAAATGAAGTC | CCTGCTCTTGTCTTGTTA |
| RCCR1 | CGCATTTCCTCATGGAATTT | CTTCTCACGCTGTTTGTCCA |
| SGR | AGGGGTGGTACAACAAGCTG | GCTCCTTGCGGAAGATGTAG |
| porA | ATCACCAAGGGCTACGTCTC | GAGTTGTTGTTCCAGCTCCA |
| rbcL | GTTGAAAGGGATAAGTTGA | AATGGTTGTGAGTTTACG |
| rbcS | TCATCAGCTTCATCGCCTAC | ACTGGGAACACACGAAACAA |
| psbA | AAGTTTCTCTGATGGTATG | ATAGCACTGAATAGGGAA |
| psbS | GCTGTTCGGCTTCACCAAGG | ACGCCGGTCTCGATGTTGA |
| cab2R | GTTCTCCATGTTCGGCTTCT | GACGAAGTTGGTGGCGTAG |
| CHLI | AGTAACCTTGGTGCTGTG | AATCCATCAACATTCAACTCTG |
| CHLD | GGAAAGAGAGGGCATTAG | CAATACGATCAAGTAAGTGTT |
| NPH1a | CTTTTAGAGGAAAAACGAGGCAACG | TGTAGCAACCTATACATTAGCTGCC |
| CAO | AGGAATAAAACCAGGCTGCTCT | CATTCAAGACCTTCTCAGCAAAA |
| HEMA | AAGCAATGGAGGCCCAAA | TCCGAAGCCCTGATCCTGTC |
| OsSAMS1 | CAAGAAAGGCGGCAACGGA | AGGCAGAAGGCTTCTCCCA |
| OsNAP | GCCAAGAAGCCGAACGGTT | CCCCATGTTAGAGTGGAGCAG |
| OsNYC3 | TGGTTTCTTCCAGCCACTCC | TTGGCCGAGTGAGCTTTGC |
| OsABA8ox1 | AAGCTGGCAAAACCAACATC | CCGTGCTAATACGGAATCCA |
| OsABA8ox2 | CTACTGCTGATGGTGGCTGA | CCCATGGCCTTTGCTTTAT |
| OsABA8ox3 | AGTACAGCCCATTCCCTGTG | ACGCCTAATCAAACCATTGC |
| OsNCED1 | ACCATGAAGTCCATGAGGCT | TCTCGTAGTCTTGGTCTTGG |
| OsNCED2 | ATGGAAACGAGGATAGTGGT | CTTATTGTTGTGCGAGAAGT |
| OsNCED4 | ATCTCCTTCTCCCTCCTCCCA | TCGCACCCTGCTTGATCTTGC |
| KO2 | TGAAGTAGCCAAGGAGGCGA | CGCTGATTGCGACCATACTTT |
| CPS | GCGTGCATTTTCGAACCAA | TTGGCCAGCACTGACACTCT |
| OsGA20ox2 | GGGAGGGTGTACCAGAAGTACTG | GGCTCAGCTCCAGGAGTTCC |
| OsGA3ox2 | TCTTCTCCAAGCTCATGTGGT | AACTCCTCCATCACGTCACAG |
| OsGA2ox1 | TGACGATGATGACAGCGACAA | CCATAGGCATCGTCTGCAATT |
| OsGA2ox3 | TGGTGGCCAACAGCCTAAAG | TGGTGCAATCCTCTGTGCTAAC |

**Supplementary Dataset**

Supplementary Dataset 1. Differentially expressed genes (DEGs) between *yl3* and WT in transcriptome analysis
